# Molecular analysis and computational modeling reveal temporally separable responses triggered by DENV-induced soluble factors in endothelial cells

**DOI:** 10.1371/journal.pone.0354877

**Published:** 2026-07-31

**Authors:** Jenny Paola Alfaro-García, Julieta María Ramírez-Mejía, Paola Rojas-Estevez, Diego Alejandro Álvarez-Díaz, Geysson Javier Fernández, Carlos Alberto Orozco-Castaño, Boris Anghelo Rodríguez-Rey, Juan Carlos Gallego-Gómez, Miguel Vicente-Manzanares

**Affiliations:** 1 Grupo Medicina de Translación—Facultad de Medicina, Universidad de Antioquia, Medellín, Colombia; 2 Grupo de Biología del Cáncer—Instituto Nacional de Cancerología, Bogotá, Colombia; 3 Grupo de Genómica de Microorganismos Emergentes—Dirección de Investigación en Salud Pública, Instituto Nacional de Salud, Bogotá, Colombia; 4 Grupo de Investigación y Desarrollo en Vacunas y Biológicos Estratégicos en Salud Pública—Dirección de Producción, Instituto Nacional de Salud, Bogotá, Colombia; 5 Biología y Control de Enfermedades Infecciosas—Corporación Académica para Estudio de Patologías Tropicales, Universidad de Antioquia, Medellín, Colombia; 6 Grupo de Fundamentos y Enseñanza de la Física y los Sistemas Dinámicos—Universidad de Antioquia, Medellín, Colombia; 7 Molecular Mechanisms Program, Centro de Investigación del Cáncer, Instituto de Biología Molecular y Celular del Cáncer, Consejo Superior de Investigaciones Científicas (CSIC)—University of Salamanca, Salamanca, Spain; Instituto Nacional de Salud Publica Centro de Investigaciones sobre Enfermedades Infecciosas, MEXICO

## Abstract

Dengue virus (DENV) represents a growing global health challenge with billions of people at risk. Severe Dengue (SD), a complication of DENV infection that involves generalized hemorrhage, is driven, at least in part, by endothelial dysfunction. Endothelial dysfunction refers to increased permeability due to inflammation, mechanical injury and/or modification of the genetic program of endothelial cells. Previous work showed that exposure of endothelial cells to conditioned media from DENV-infected cells (CMDV) increased permeability and cellular stiffness, repressed endothelial markers and induced mesenchymal genes. However, the generality, extent, mechanism and ultimate impact of these events in the onset of SD remain elusive. Here, we integrate analysis from in vitro treatment of endothelial cells with media containing UV-inactivated DENV with computational modeling to investigate the key features of CMDV-induced endothelial alterations and their potential impact on endothelial dysfunction. We found that CMDV increased *SNA1* and *CDH2* expression, while suppressing endothelial genes *OCLN* and *CDH5*. Global transcriptomics analysis revealed that CMDV triggered a transient pro-inflammatory response, followed by induction of selected tissue repair genes and matrix remodeling. A non-directed asynchronous network model (NDAM-CMDV) identified IL6 and FN1 as central nodes of DENV-induced endothelial trans-differentiation, providing new molecular insights that predict the evolution of the disease and identify potential therapeutic targets.

## 1. Introduction

Dengue virus (DENV) is currently the most prevalent arbovirus in the world. The largest outbreak of dengue was recorded between 2023 and 2024, underscoring its growing prominence as a global threat to human health. Estimates suggest that half of the human population may be at risk [1–3]. Of particular concern is the progression of dengue to severe dengue (SD), a rare but life-threatening condition characterized by generalized hemorrhage. There is no specific treatment for SD, only palliative care [4,5].

Hemorrhage in SD is due to several, poorly characterized, factors. A central feature of SD is endothelial dysfunction, which seems caused by activation of the innate immune response; expression of pro-inflammatory mediators that activate endothelium; antibody-dependent enhancement of viral infection [6,7]; and/or extracellular matrix (ECM) remodeling in response to cytopathic damage [8]. Notably, SD typically arises during the defervescence stage [4], when viremia is declining [9]. Conversely, viral proteins, e.g., NS1, and DENV-induced host factors such as IL-6, TNF-α, NF − κB, TGF-β and others persist during this stage [10–12].

The expression and effect of those soluble factors can be recapitulated in vitro using conditioned media from DENV-infected cells (CMDV). In this model, media are irradiated with UV light, which inactivates the virions but does not affect viral proteins and host factors. Previous reports have indicated that incubation of epithelial and endothelial cells with CMDVs activate migratory pathways involving c-Abl and RhoA [13–15]. Additionally, CMDVs induce a transient reorganization of the cytoskeleton. They also decrease the expression of selected endothelial markers such as ZO-1 and VE-Cadherin [14,16], while increasing expression of mesenchymal markers such as Snail (gene *SNA1*), Twist1 (*TWIST1*), α-SMA (*ACTA2*) and N-cadherin (*CDH2*) [16]. These effects were concurrent with increased endothelial permeability, which is partially reversed by treatment with imatinib [14].

These data strongly suggest that the host soluble factors induced by DENV infection trigger increased permeability due to the weakening of cell-cell junctions and the acquisition of migratory features. These changes are also consistent with a process known as Endothelial-to-Mesenchymal Transition (EndMT) [14,16]. EndMT is defined as a reversible trans-differentiation process that occur in healthy and pathological processes, such as wound healing and cancer, respectively [17]. Interestingly pathogens such as bacteria [18–20] and viruses [21–23] may also trigger EndMT. It is important to note that EndMT is not a binary switch in which cells remain completely endothelial or turn completely mesenchymal. Instead, different studies highlight the existence of multiple intermediate stages [24–26]. In the present study, we pose that CMDVs may induce changes in endothelial cells that recapitulate specific aspects of EndMT. Such events would constitute a suitable explanation for the conspicuous absence of significant post-mortem evidence of inflammatory damage in patients who succumbed to SD [27–29]. This confirms previous studies from the group that had provided partial evidence for such possibility [14,16]. However, the kinetics of the process and the potential overlap and/or synergy with the inflammatory response remain unknown. In addition to recapitulating and quantifying the effect of CMDV in selected markers of endothelial and mesenchymal lineages, global transcriptomics have revealed a genetic link to the biphasic response observed in severe dengue patients, that is, high viremia associated with elevated fever (where we observe an induction of pro-inflammatory mediators), followed by more severe symptoms during the defervescing phase, where we detect induction of endothelial plasticity. Together, these data enabled us to generate an unbiased model based on transcriptomic data from endothelial cells treated with CMDV compared to untreated cells and cells treated with TGF-β, a bona fide inducer of EndMT. Computational modeling and perturbation analysis predict a biphasic response to DENV infection in endothelial cells, characterized by an initial period dominated by the inflammatory response (when viremia is reportedly high), followed by a repair/remodeling response later on, during the defervescence stage, when the viremia is reportedly declining.

In summary, our findings provide in vitro and in silico support to the hypothesis that partial EndMT induced by soluble factors stemming from DENV infection plays a crucial role in the pathogenesis of SD, forming the conceptual basis of novel approaches that could identify critical targets to treat SD patients.

## 2. Results

### 2.1. CMDV induces expression of mesenchymal markers and morphological alterations in endothelial cells

**Fig 1:**
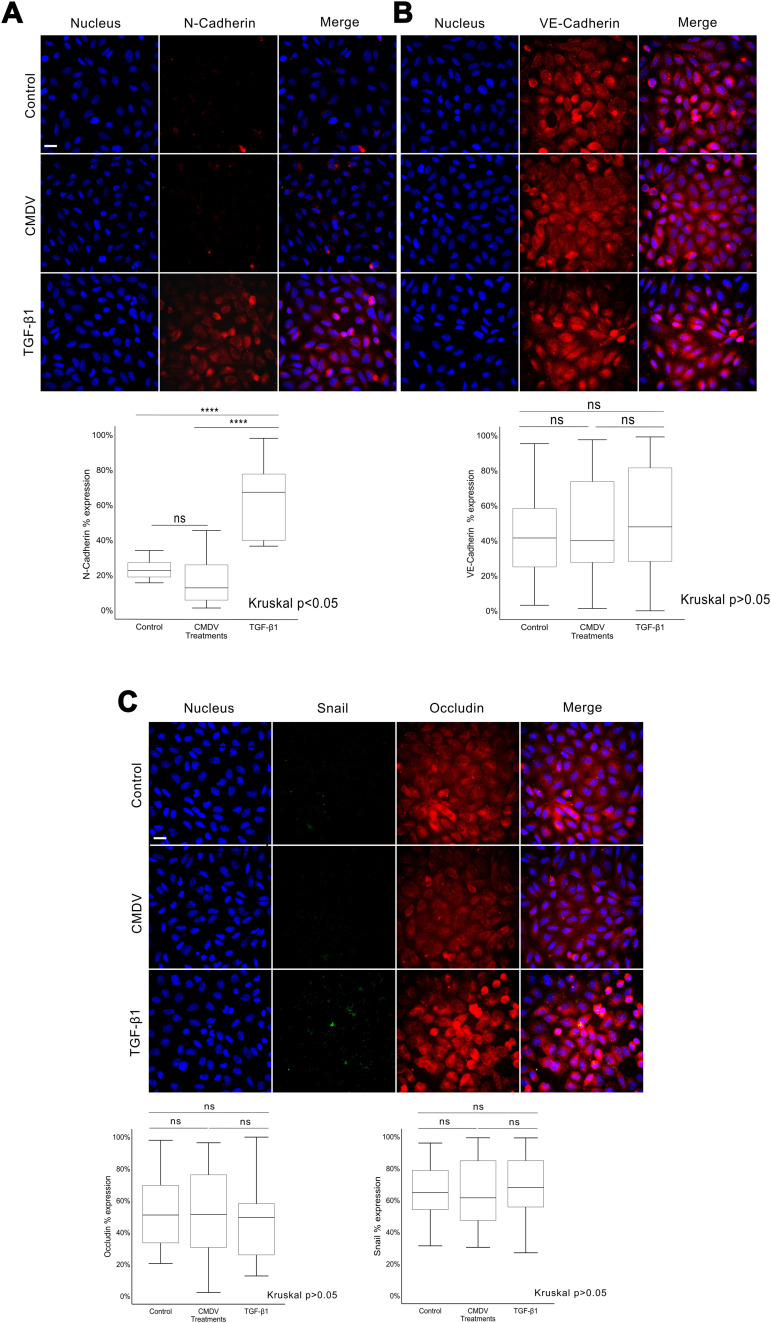
Effect of CMDV and TGF-β1 on the expression of selected endothelial and mesenchymal markers in HMEC-1 endothelial cells at 48h. Representative images of HMEC-1 cells treated as indicated and stained for DNA (DAPI) and N-Cadherin (A), VE-Cadherin (B) and occludin and Snail (C). In overlays, red is N-cadherin (A), VE-cadherin (B) or occludin (C) as indicated, and green is Snail (C only); blue represents DAPI staining in all overlays. Scale bar = 20 µm. Bottom, quantitative analysis of images as in top. See Material and Methods for details. Data are presented as mean ± SEM from >100 fields examined in three independent experiments. Statistical tests are indicated, and significance is as follows: *p < 0.05, **p < 0.01, ***p < 0.001, ****p < 0.0001.

**Fig 2:**
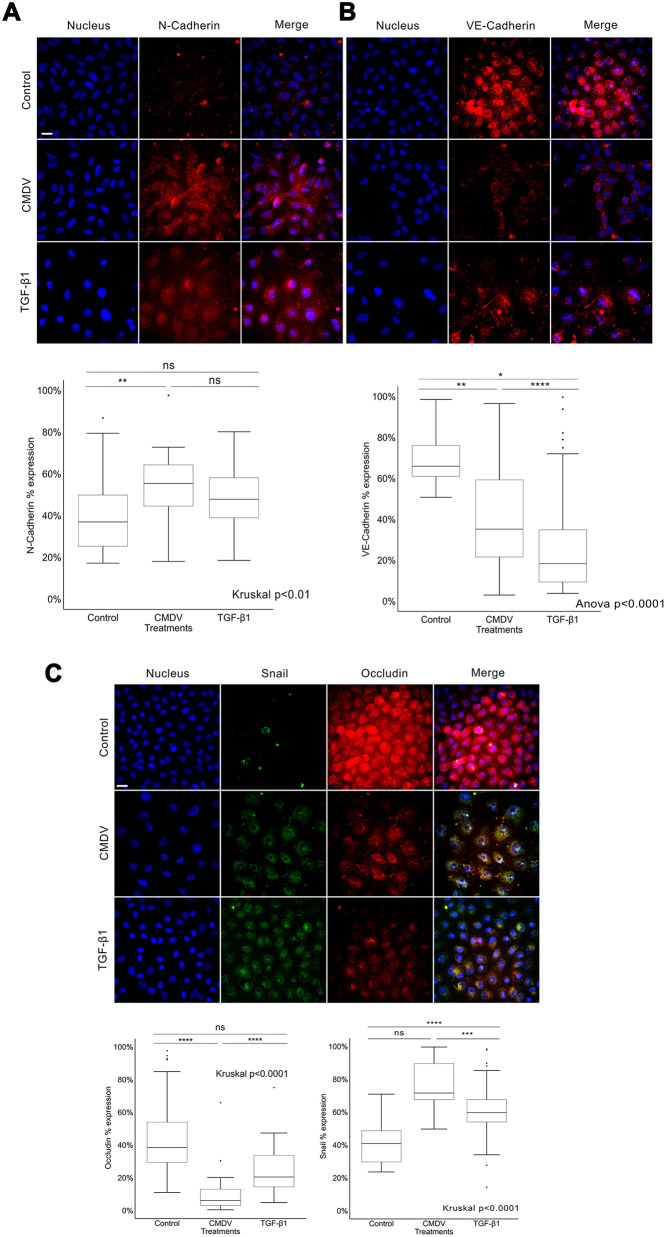
Effect of CMDV and TGF-β1 on expression of selected endothelial and mesenchymal markers in endothelial cells at 120h. Representative images of HMEC-1 cells treated as indicated and stained for DNA (DAPI) and N-Cadherin (A), VE-Cadherin (B) and occludin and Snail (C). In overlays, red is N-cadherin (A), VE-cadherin (B) or occludin (C) as indicated, and green is Snail (C only); blue represents DAPI staining in all overlays. Scale bar = 20 µm. Under the microphotographs, quantitative analysis of images as in top. See Material and Methods for details. Data are presented as mean ± SEM from >100 fields examined in three independent experiments. Statistical tests are indicated, and significance is as follows: *p < 0.05, **p < 0.01, ***p < 0.001, ****p < 0.0001.

To evaluate the effect of CMDV on endothelial trans-differentiation, HMEC-1 (endothelial) cells were exposed for 48 hours or 120 hours to CMDV or 5 ng/mL TGF-β1. The latter was used as a positive control that induces EndMT [30]. In agreement with previous results, CMDV had no cytopathic effect [16]. At a protein level, CMDV neither induced expression of N-cadherin (*CDH2* gene) nor reduced expression of VE-cadherin (*CDH5* gene) at 48 hours (Fig 1A-B). However, N-cadherin induction and VE-cadherin reduction by CMDV were significant after 120h (Fig 2A-B). Similarly, CMDV did not affect Snail or occludin expression after 48h (Fig 1C), but reduced occludin and increased Snail expression significantly after 120h (Fig 2C). Conversely, N-cadherin expression was induced more rapidly (48h) by TGF-β1 (Figure 1A-B). Interestingly, TGF-β1 slightly increased VE-cadherin expression (Fig 1B) and had no significant effect on the expression of occludin and Snail (Fig 1C) at 48h. However, TGF-β1 reduced VE-cadherin and occludin expression and increased Snail levels after 120h, as expected (Fig 2B), suggesting that the loss of endothelial markers and the acquisition of mesenchymal markers during TGF-β1-induced EndMT is not a linear process.

Imatinib, a c-Abl inhibitor previously shown to counter some of the effects of exposure to CMDV [14,16], curbed the increased expression of Snail and N-cadherin and prevented the decrease of VE-cadherin and occludin (S1 and S2 Figs). However, imatinib-treated cells did not recover their morphological integrity, likely due to direct effects of the inhibitor on the actin cytoskeleton [31].

### 2.2. CMDV transiently enhances pro-inflammatory gene expression

We next performed RNAseq of CMDV- and TGF-β1-treated endothelial cells compared to untreated cells, in conditions similar to those in Figure 1-2. 48 hours post-treatment, we found 98 DEGs (differentially expressed genes), 75 upregulated and 23 downregulated in response to CMDV. After 120 hours of incubation with CMDV, the total DEG number increased to 742, 401 upregulated and 341 downregulated. Conversely, TGF-β1 treatment for 48 hours produced 840 DEGs, 401 upregulated and 439 downregulated. After 120 hours, we found 1506 DEGs, 827 upregulated and 679 downregulated.

**Fig 3:**
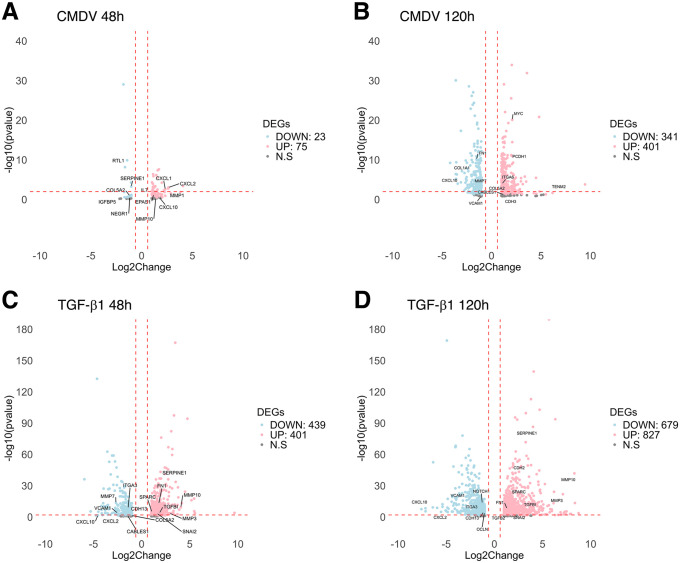
Volcano plots depicting DEGs at 48h and 120h after exposure to CMDV (A-B) and TGF-β1 (C-D). See Material and Methods for technical details on sample collection and analysis. In the graphs, red means upregulated and blue means downregulated. Grey represents no significance (genes are located beyond the red dotted lines, denoting the significance threshold). Experiment is representative of three independent replicates.

Analysis of DEGs (differentially expressed genes) expressed by HMEC-1 cells in response to CMDV revealed that the treatment triggered two distinct gene expression patterns: an initial pro-inflammatory response at 48 hours, including less than 100 genes, for example IL-6, CXCL1, CCL1/6 and several growth factors (Fig 3A and S3 Fig). At 120h, we observed an endothelial tissue repair program containing over 800 DEGs, including repair genes such as MYC, TENM2 and specific forms of collagen, e.g., COL5A2 (Fig 3B and S4 Fig). This response is markedly different from that induced by TGF-β1 at 48h (Fig 3C and S5 Fig), which induced over 800 DEGs including EndMT-related genes and cell junctions. At 120h, > 1500 DEGs that included EndMT and cell cycle-related proteins (Fig 3D and S6 Fig). This indicates that the effects of CMDV are not solely due to TGF-β1 induced during DENV infection, as seen in peritoneal macrophages [32].

As expected, TGF-β1 did not trigger the expression of inflammatory genes at 48h, whereas CMDV mainly induced inflammatory cytokines such as CXCL1, CXCL2, CCL2 and IL7; other inflammatory mediators such as IRAK3 and NRP2; and metalloproteases such as MMP1 and MMP10. Pharmacological interaction analysis (DGIdb) identified potassium alum as a potential modulator of IL-7 in this phase (S1 Table). TGF-β1 triggered mesenchymal genes such as *SNAI2* (Slug protein), *SMAD7*, *TGF-β1*, *ITGA2* and others, while inflammatory markers such as *CXCL6*, *CXCL8* or *STAT4* were repressed. Interestingly, additional pharmacological potential targets emerged in this phase, including Anakinra, which is an antagonist of IL1R2 (S1 Table). Conversely, 120h of CMDV treatment induced a response focused on cell repair, including genes such as MYC*.* At 120h, most of the inflammatory genes induced at 48h were repressed (Figure 3B).

On the other hand, 120h exposure to TGF-β1 promoted expression of mesenchymal markers such as *TGF-β1/2/3*, *SNAI2*, *FN1* and *VCL*, among others (Fig 3D). Interestingly, both TGF-β1 and CMDV modulated genes involved in ECM remodeling, including collagens, integrins, and metalloproteases. This suggests a potential reorganization of cell–cell and cell–matrix interactions, directing endothelial cells toward a more mesenchymal-like phenotype.

**Fig 4:**
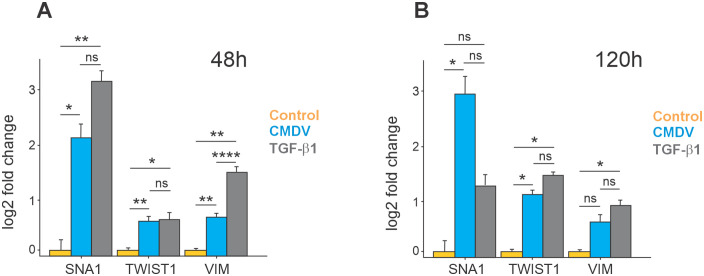
Semi-quantitative PCR of SNA1, TWIST1, and VIM gene expression at 48 hours (A) and 120h (B) post-treatment with CMDV and TGF-β1. Data is relative to untreated controls. Results are presented as mean ± SEM of three independent experiments, and statistical significance was determined using T test or Wilcoxon test (p < 0.05). Significance between samples against untreated control is shown on top of each comparison. *p < 0.05, **p < 0.01, ***p < 0.001, ****p < 0.0001.

We validated the expression of *SNA1* (encoding Snail protein), *TWIST1* (Twist1) and *VIM* (vimentin) mRNA observed in the RNAseq analysis by RT-qPCR, using *ACTB* as housekeeping gene. The results revealed that CMDV increased the transcription of those three genes after 48 hours (Fig 4A) and 120 hours of treatment (Fig 4B).

We then performed GO and pathway enrichment analysis on the RNAseq data. The main biological process (BP) induced after 48h treatment with CMDV was immune response (S7 Fig). Molecular functions (MF) clustered around receptor ligand activity and cytokine pathways, but the Cellular component (CC) was not significant. In contrast, the main biological processes triggered by 48h treatment with TGF-β1 were cellular migration and differentiation, with cellular components (CC) involved in cytoskeleton-extracellular matrix and adhesion. Interestingly, the major alterations to MF induced by TGF-β1 at 48h were calcium ion binding and sensory perception of mechanical stimulus (S8 Fig).

The main BPs induced by 120h treatment with CMDV were mitosis, including mitotic sister chromatid segregation, and cellular differentiation (S9 Fig). Meanwhile, CC were linked to cytoskeleton and cellular adhesion, with emphasis on focal adhesions (S9 Fig). This pattern was similar to that induced by TGF-β1 at 120 hours (S10 Fig). The CC and MF induced by 120h of TGF-β1 were associated with cellular morphology changes involving the cytoskeleton, tubulin binding and related components. In summary, CMDV induces an initial pro-inflammatory response that turns into an endothelial repair response at later time points, whereas TGF-β1 purely induces trans-differentiation with a late repair response that is similar to that promoted by CMDV.

### 2.3. Common cellular alterations induced by CMDV at 48h and 120h

The endothelial response induced by CMDV at 48 hours is different from that at 120 hours, although some common genes were identified at both timepoints. This suggests potential biological processes linking the two cellular responses associated to the effects of soluble factors on the cells over time. To assess this hypothesis, the DEGs from CMDV at both 48 hours and 120 hours (S2 Table) were used to create a protein-protein network for each time-point. At 48 hours, four main clusters were identified: one was disconnected from the others, and related to cellular differentiation, while the other three were interacting with each other and were associated with the immune response (S11 Fig). At 120 hours (S12 Fig), 57 clusters were found, including large clusters that control cell cycle progression (S12 Fig, **dashed red bo**x). Additionally, one cluster was associated with cytokine signaling and several neighbor clusters were involved in ECM deposition and cell migration processes (S12 Fig, **dashed blue ellipse**). These observations were made using two independent cluster enrichment tools, StringdB and Enrichr.

To evaluate the possible connection between the three clusters identified at 48 hours and the cytokine signaling cluster along with its neighbors at 120 hours, a new interactome was created using the upregulated and downregulated genes with a p-value <0.05 in Cytoscape and StringDB. The merged network (S13 Fig) confirmed the connection between the two cellular responses, with four overlapping genes. The average local clustering coefficient (0.7) indicated that these interactions were not random. Notably, IL6 and FN1 emerged as central nodes. Furthermore, the MCL algorithm from String DB revealed additional clusters in the merged network. These clusters could be grouped into three major biological processes: immune/inflammatory response (cytokine-mediated signaling pathway), cell migration (positive regulation), and differentiation (neural crest cell migration). These processes were highly significant, with a False Discovery Rate (FDR) <6.76e-07, although the strength of the signals varied. Molecular functions were consistent with immune functions (growth factor binding, cytokine activity, and CXCR chemokine receptor binding, with high signal-to-noise ratio, strength and an FDR < 3.5e-07). Cellular component analysis identified components involved in cellular migration and differentiation (endoplasmic reticulum lumen, collagen-containing extracellular matrix, and extracellular matrix, also with a high signal-to-noise ratio, variable strength, and FDR < 2.5e-09).

Overall, these in silico observations were consistent with cellular and molecular assays, in which we observed alterations in markers related to morphological and cellular plasticity. Such consistency allowed us to select this network for further modeling.

### 2.4. Building a non-directed asynchronous model of the effect of CMDV on endothelial cell behavior (NDAM-CMDV)

**Fig 5:**
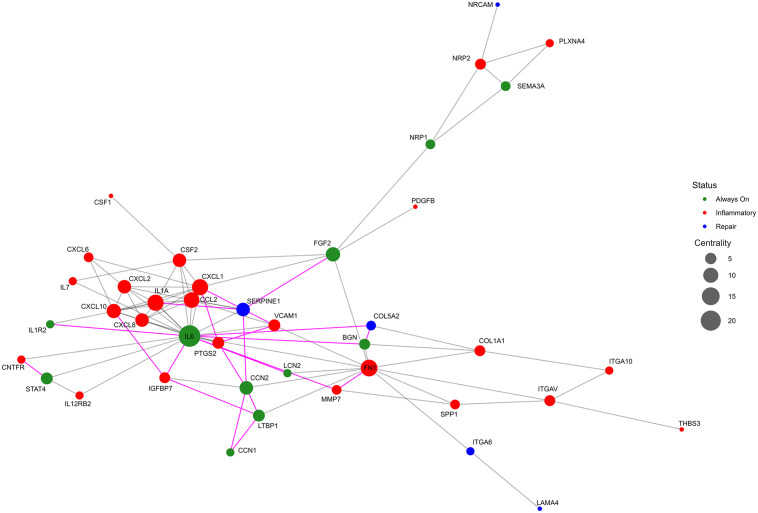
Graphic representation of the non-directed model depicting the effect of CMDV. The network depicts the nodes found in the transcriptomic analysis of CMDV-treated cells. Nodes are color-coded as inflammatory only (red), reparative only (blue) and always on (green). The size of the nodes represents centrality (node size) as indicated, with focus on IL6 and FN1. Gray lines represent edges. Magenta lines represent new interactions.

The interactome described earlier was used to formalize a set of Boolean rules (Supplementary Information, Table A) regarding the fluctuation in expression and connections, mostly based on bibliographic data due to the scant experimental data available (Fig 5). This section is described in technical detail in the Supplemental Section. Briefly, the resulting network consists of 41 nodes and 99 edges. Centrality analysis (S3A Table and Fig 5) identified IL6 and FN1 as key nodes, with other relevant nodes were CXCL1, IL1α, FGF2, and CCL2. Other network metrics are shown in S3B Table. Since the ultimate goal of this model is enable simulations that reproduce the real situation, we implemented thresholds and modulators that modify the network generally or locally [33]. The number of iterations that would be required to reach a minimum threshold of FN1 degradation were determined using a first-order modified model with regular catabolic degradation rate [34]. FN1 required 23 iterations to reach the minimum threshold. The maximum thresholds and modulators are 24, establishing 23 FN1-dependent interactions. This was used to induce changes in specific nodes. The only exception was IL1R2, which required a threshold of 22 iterations to remain in the model.

Additional regulators that influence the network in initial stages are defined by the transcription, translation and/or metabolic synthesis rates. For modeling, transcription and translation are assumed to be rapid and comparable to each other [35]. Conversely, metabolic synthesis involves multiple steps of different durations. This is best illustrated by VCAM1, an adhesive receptor produced in response to inflammation [36,37]. This introduces an asynchronous behavior component in the system, which better simulates the experimental results and rounded up a new non-directed asynchronous model of the effect of CMDV on endothelial cells (NDAM-CMDV).

### 2.5. NDAM-CMDV robustness, accuracy and attractors

**Fig 6:**
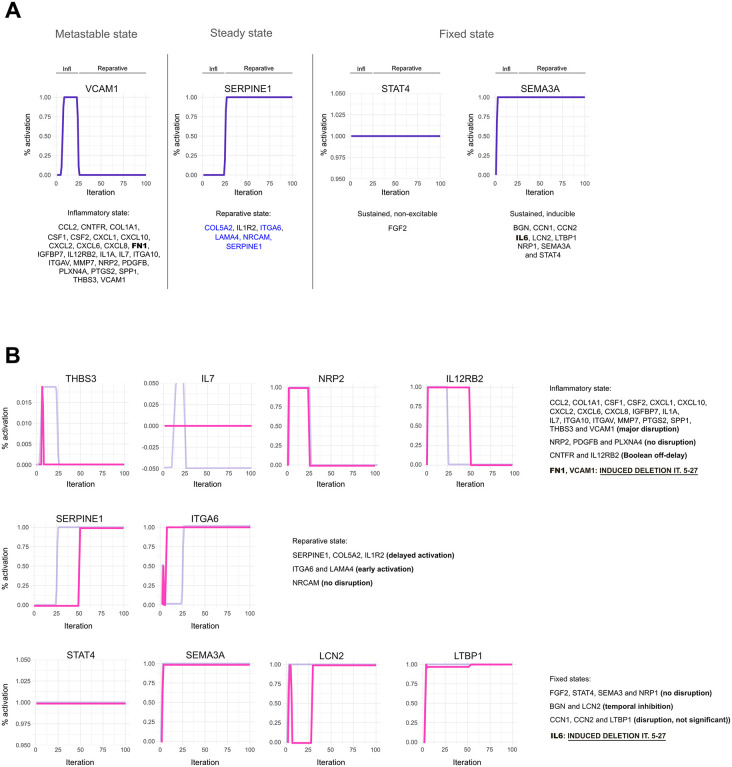
Model simulations predict gene responses in response to CMDV. A. Simulations of the nodes illustrated in Figure 5 display four main behaviors: inflammatory nodes are activated from the beginning of the simulation until approximately iteration 25, decaying thereafter (metastable state). Reparative genes are induced around iteration 25 and remain elevated for the rest of the iterations (steady state). Some genes, e.g., STAT4 and FGF2 do not become modified in response to stimulation (fixed, non-responsive), whereas other genes are induced early and remain elevated over the entire course of the simulation. B. Simulation of the effect of knocking out FN1, IL6, and VCAM1 nodes. Pale magenta lines represent the state of each node in untreated conditions (as in A). Steady state is reached after 50 iterations.

Next, we examined NDAM-CMDV accuracy and robustness. We performed 25000 simulations, each consisting of 100 iterations with asynchronous activation (Fig 6 and S4 Table). NDAM-CMDV exhibited the expected behavior: pro-inflammatory nodes were activated during the first 25 iterations and then turned off; the remaining 75 iterations corresponded to the reparative/plasticity response.

The activation percentage, media, and standard deviation from each node was highly predictable because they exhibit high activation but low variance (STAT4, FGF2, IL6, NRP1, SEMA3A, BGN, and LCN2). This means these nodes are consistent across the entire system. Another subgroup of nodes displays low means and variance (IL1A, CCL2, CSF2, CSF1, and IL7). Finally, another high predictability group includes those with high means and medium variance (CCN1, CCN2, LTBP1, and IL1R2), indicating that they are activated and tend to be consistent. However, other nodes tend to be more unpredictable due to their high variance and average means (SERPINE1, COL5A2, NRCAM, LAMA4, and ITGA6), or high variance and low means (IL12RB2, NRP2, CNTFR, PDGFB, FN1, and PLXNA4). Every node (gene) fell into four possible categories as shown in Fig 6A: i) induced during the inflammatory response (iterations 1–25); ii) induced only during the reparative response (iterations 26–100); iii) non-responsive; and iv) induced during the inflammatory response and sustained during the reparative phase.

On the other hand, we also determined the system attractors (S5 Table). The analysis identified them using various approaches, including synchronic and asynchronous methods, thereby identifying synchronic and asynchronous attractors. In the first two analyses, attractors were consistent (STAT4, IL6, FGF2, NRP1, SEMA3A, LCN2, BGN, ITGA6, LAMA4, LTBP1, CCN1/2, SERPINE1, IL1R2, COL5A2, and NRCAM), suggesting that these nodes influence the dynamic behavior of the system. However, this analysis identified 31 highly recurrent nodes. This strongly indicates that the model may be misaligned. Nevertheless, eight attractors (IL6, SEMA3A, LCN2, BGN, LTBP1, CCN1, CCN2, and IL1R2) were common in all the analyses. These indicate that the network has a moderate degree of complexity, high stability, and predictability, which are strong indicators of robustness. Additionally, the accuracy of the CMDV model compared to the experimental data is good, indicating its potential usefulness to study endothelial dysfunction in dengue using a cellular approach.

### 2.6. NDAM-CMDV can be used to predict endothelial responses to Dengue infection in silico

To test the performance of NDAM-CMDV under different conditions, we introduced observer-directed perturbations involving specific nodes in iterations 5–27. In the first perturbation, we deleted PTGS2 (S14A Fig). PTGS2 suppression caused a partial inhibition of the pro-inflammatory response, abrogating the induction of CCL2, CSF1, CSF2, CXCL10, CXCL8, IGFBP7, IL1A, IL7, and VCAM1. It also decreased the levels of CXCL10, CXCL8, IL1A, CSF1/2, IGFBP7, and IL1R2 (S12 Fig and S6 Table).

Next, we deleted IL6 (S14B Fig and S7 Table). This perturbation prevented the induction of CCL2, COL1A1, CXCL1, CXCL2, CXCL10, CXCL6, CXCL8, IGFBP7, IL1A, IL7, ITGA10, ITGAV, PTGS2, VECAM1, THBS3, and SPP1, while CSF1, CSF2, and MMP7 displayed a brief activation that declined rapidly. Additionally, IL6 deletion impairs expression of BGN, LCN2, IL1R2, SERPINE1, IL12RB2, and CNTFR.

Next, we deleted BGN and LCN2 simultaneously (data not shown). We found that this perturbation does not greatly affect the system. The main defect was a delay in the induction of COL5A2, IL1R2 and SERPINE2 until iteration number 50, explaining why their activation percentage decreased. Meanwhile, IL12RB2 and CNTFR increased because they remain present in the system until iteration 50.

We also tested the effects of a triple deletion scenario affecting IL6, FN1, and VCAM1 (Fig 6B and S8 Table). Simulations predicted a complete inhibition of CCL2, CXCL10, IGFBP7, IL1A, and IL7 expression, with brief activation of COL1A1, CSF1, CSF2, CXCL1, CXCL2, CXCL8, CXCL6, FN1, ITGA10, ITGAV, PTGS2, THBS3, MMP7, and SPP1. The triple knockout disrupted the behavior of BGN and LCN2. Their expression outside the system recovered after iteration 27. CCN1, CCN2, and LTBP1 displayed decreased activation that is restored after iteration 27, while CNTFR, IL12RB2, IL1R2 and SERPINE1 behaved as reported when only IL6 was deleted (S14B Fig and S7 Table). Conversely, ITGA6 and LAMA4 activated faster than in control conditions.

In addition, we deleted FN1 but overexpressed FGF2 (S14C Fig and S9 Table). CCL2, IL7, and VECAM1 were inhibited, whereas COL1A1, CSF1, CSF2, ITGA10, MMP7, SPP1, and THSB3 displayed a brief activation. Additionally, CCN1/2, IL1R2, ITGAV, and LTBP1 were modestly reduced at the beginning, recovering thereafter. Similar to the previous simulation, ITGA6 and LAMA4 were activated early.

**Fig 7:**
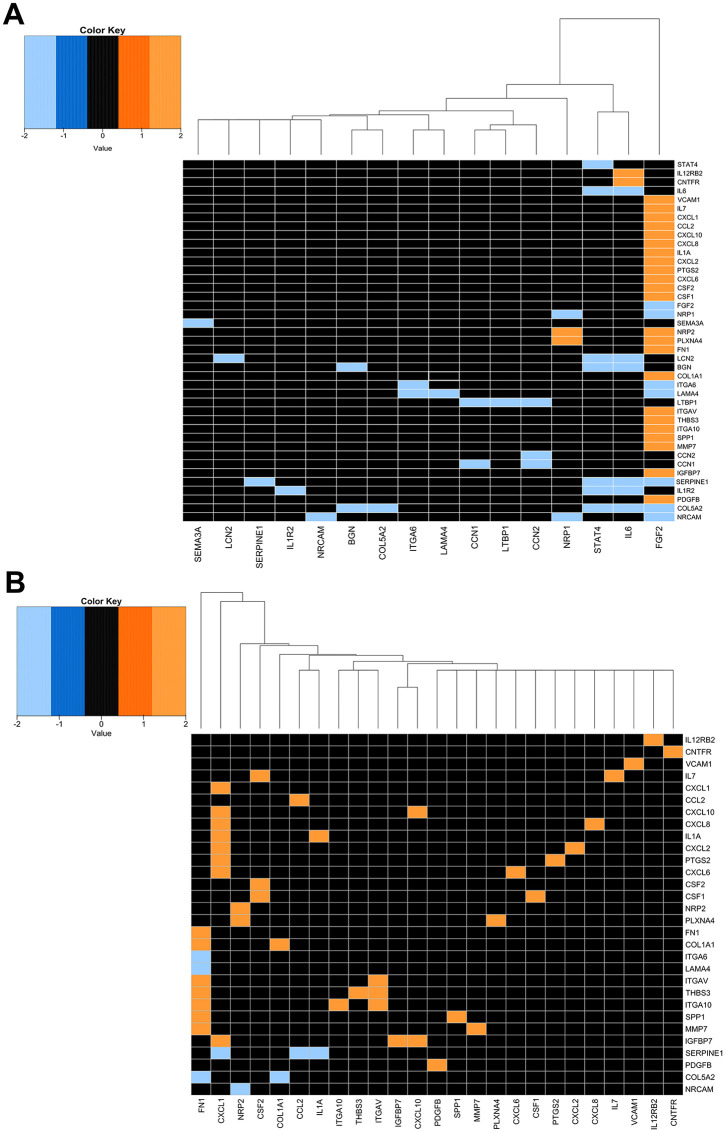
Hierarchical clustering of perturbation analysis of the effects of a single mutated node in the CMDV network. (A) Knockout of each node and their closeness. FGF2 knockout increases the expression of pro-inflammatory nodes. (B) Overexpression of each node and their closeness. FN1 enhances expression in some pro-inflammatory nodes such as MMP7. This is similar for CXCL1, but its effect is local and modifies the CXCL cluster.

Finally, we analyzed the effect of a single node knockout (Fig 7A) and single node overexpression (Fig 7B). FGF2 knockout increased the levels of VCAM1, IL7, CXCL1, CCL2, CXCL10, CXCL8, IL1A, CXCL2, PTGS2, CXCL6, CSF1, CSF2, NRP2, PLXNA4, FN1, COL1A1, ITGAV, THBS3, ITGA10, SPP1, MMP7, IGFBP7, and PDGFB, while it decreased the levels of NRCAM, COL5A2, LAMA4, ITGA6, and NRP1. Additionally, we found that most nodes exhibit a similar expression profile, except for NRP1.

Conversely, overexpression of FN1 increases MMP7, COL1A1, ITGAV, THBS3, ITGA10, and SPP1, while reducing COL5A2. Additionally, CXCL1 enhances IGFBP7, CXCL6, PTGS2, CXCL2, IL1A, CXCL8, and CXCL10, while decreasing PDGFB. Most nodes exhibit similar expression levels, although there are slight differences. Interestingly, NRP2 and CSF2 share similar expression patterns, while FN1 and CXCL1 are related, yet display completely different expression profiles compared to the other nodes. Increasing the sensitivity of the analysis decreased variation and masked mutational effects per node (S15 Fig).

## 3. Discussion

Soluble factors released during DENV infection induce a transient and partial EndMT-like state in endothelial cells. In our model, exposure to CMDV caused morphological changes, intercellular junction disruption, and upregulation of some EndMT markers, leading to increased endothelial permeability [16]. Notably, this response was biphasic and reversible: at 48 hours, CMDV-exposed cells displayed a strong pro-inflammatory profile, whereas by 120 hours they had shifted toward an endothelial repair and angiogenic profile, similar to what has been reported for EMT [38]. This pattern (an initial inflammatory surge followed by a possible reparative phase) may explain why endothelial dysfunction in severe dengue is often transient and does not always lead to irreversible vascular injury [9,11,12]. We posit that CMDV triggers an acute, partial EndMT that compromises the endothelial barrier during the peak infection period that may be naturally resolved in the absence of additional inflammatory stimuli and/or additional factors that sustain inflammation or exacerbates the repair response debilitating endothelial cell junctions. Importantly, the four serotypes of dengue produce the same disease; the difference is in the onset of the symptoms or their intensity [39–42]. While disease produced by any serotype can evolve to SD, DENV2 has been extensively associated with this complication and a higher mortality rate [43].

These findings align with and extend previous observations of dengue pathogenesis. Prior studies have shown that pro-inflammatory cytokines and the viral NS1 protein can disrupt endothelial junctions and increase vascular permeability [6,10–12]. Our results support this framework and further indicate that other soluble mediators present during infection (beyond NS1, which is not completely absent from media in similar conditions [44]) also play a critical role in endothelial dysfunction.

The analysis of dengue virus–induced cytokines in our model reveals a strong pro‑inflammatory signature—dominated by IL‑6, CXCL1, CXCL2, CXCL8, and CCL2—which mirrors the central role these mediators play in endothelial activation and vascular barrier disruption. Although NS1 was not directly measured in this specific set of experiments, its presence in DVC is expected due to its well-documented secretion and persistence during DENV infection [10]. Secreted flavivirus NS1, including DENV2 NS1, can directly alter endothelial permeability in vitro in a cell type-dependent manner [45], experimental evidence suggests this effect is largely independent of inflammatory cytokines in HMEC-1 cells, as they do not produce TNF‑α, IL‑6, or IL‑8 upon NS1 exposure alone. Instead, NS1 primarily acts via the activation of glycocalyx-degrading enzymes such as sialidases, cathepsin L, and heparanase [46].

Clinically, the relationship between specific cytokines and severe dengue remains complex. While reports for TNF‑α, IL‑1β, and CXCL8/IL‑8 are inconsistent, IL‑6 levels are consistently associated with a pathogenic role in patients [10,11]. Furthermore, high levels of NS1 in plasma do not always correlate temporally with vascular leakage in vivo, often appearing after NS1 levels have declined [46]. Our model suggests that the synergistic effect of infection-induced pro-inflammatory cytokines and NS1 may act through converging pathways to promote junctional remodeling and vascular dysfunction [10].

Unlike the permanent EndMT seen in chronic fibrotic diseases or cancer [17,47], the partial EndMT-like changes induced by CMDV appear incomplete and transient. Endothelial cells acquired some mesenchymal traits (e.g., elevated Snail, N-cadherin, vimentin) without fully losing their endothelial identity [48–52]. This suggests a partial EndMT that may facilitate immune cell recruitment, rather than leading to irreversible mesenchymal conversion. In fact, the early secretion of chemokines (CXCL1, CXCL2, CCL2) by CMDV-treated cells would promote leukocyte adhesion and transmigration, consistent with cytokine-driven EndMT in inflammatory settings [53–56] and the known role of these chemokines in neutrophil recruitment [57,58].

Mechanistically, CMDV appears to activate both inflammatory signaling and mechanotransduction pathways in endothelial cells. The surge of IL-6 and other cytokines at 48 h indicates activation of canonical inflammatory cascades (e.g., JAK/STAT) that drive endothelial activation and EndMT [49,59,60]. In parallel, we observed upregulation of TWIST1 and transient focal adhesion disassembly, implicating pathways associated with shear stress responses [47–49,61]. It is possible that CMDV-induced extracellular matrix degradation and cytoskeletal remodeling provide cues resembling mechanical stress, thereby promoting a migratory, EndMT-like phenotype. By 120 h, as expression of inflammatory stimuli declines, endothelial cells upregulate genes related to cell proliferation and tissue repair [50–52,54], which may eventually lead to the normalization of the cells. Whether such return to normalcy is due to the transcriptomic changes seen in this phase or by the absence of stimulation in the media remains to be investigated.

This transient, partial EndMT-like burst – followed by eventual recovery – may be an adaptive mechanism to limit long-lasting vascular damage [24]. Computational modeling further underscores IL-6 and fibronectin (FN1) as critical drivers of the effect of the CMDV. In silico removal of either diminished the inflammatory and ECM responses. This correlates well with the crucial role of IL-6 in endothelial dysfunction [16,59] and FN1 in maintaining vascular integrity [53]. Targeting IL-6 could attenuate the early cytokine-mediated damage; indeed, IL-6 inhibitors such as tocilizumab merit exploration in severe dengue [55]. Likewise, preventing FN1-driven extracellular matrix breakdown might help preserve endothelial junctions and barrier function [53]. Another potential strategy is to stabilize endothelial cells during infection. We have shown that the tyrosine kinase inhibitor imatinib partially restored endothelial junction integrity after CMDV exposure. Imatinib has protective effects on the endothelium during DENV infection by stabilizing VE-cadherin and the actin cytoskeleton [14]. Therefore, imatinib could address both the inflammatory and mechanical aspects of dengue-induced endothelial dysfunction, potentially yielding a synergistic benefit [14,55]. Beyond these specific examples, our integrative approach provides a framework to screen additional compounds: by targeting key nodes like IL-6 or FN1 in the network, future studies can identify and test novel interventions to mitigate dengue-associated vascular leakage.

This study has identifiable limitations. The in vitro model used here, while informative, cannot fully replicate the complexity of DENV infection in vivo. In vivo, endothelial cells are influenced by interactions with immune cells and hemodynamic forces, which were absent in our cell culture system [17,26]. Second, while the effect of CMDV on vascular leakage can be inferred from previous studies, including our own [14–16], we did not measure endothelial permeability in real time. Finally, the Boolean network model is an abstraction that would benefit from incorporation of kinetic data and further experimental validation to improve its predictive power [33,56]. As it is, it is a useful hypotheses generator that provides a justifiable conceptual framework for future prediction, investigation and experimental validation in vitro and in vivo. Future studies, including animal models and patient samples, are required to confirm the transient partial EndMT-like mechanism *in vivo* and to assess the proposed therapeutic strategies under physiological conditions. In this context, identifying IL-6 and FN1 as key drivers of this process should be an experimental priority that would situate these two genes as concrete targets for therapies aimed at preserving vascular integrity in severe dengue.

In conclusion, our data and model sustain that soluble factors from dengue infection promote a reversible, partial EndMT-like mechanism in endothelial cells, linking the acute inflammatory surge to transient vascular leakage. These findings advance our understanding of dengue pathophysiology and lay the groundwork for targeted interventions to prevent life threatening vascular complications. Moreover, the concept of a regulated, transient EndMT-like process may have broader relevance for other acute inflammatory conditions involving endothelial dysfunction, such as sepsis [57,58].

## 4. Materials and methods

### 4.1. Cell lines and viral infections

Human Microvascular Endothelial Cells (HMEC-1) (ATCC Cat# CRL-3243) were maintained at 37°C with 5% CO2 in RPMI supplemented with 10% FBS, 10mM L-glutamine, 100 U/mL penicillin/100 mg/mL streptomycin (P/S), 10ng/mL Epidermal Growth Factor (hEGF), and 1 µg/mL Hydrocortisone. Cells were used up to passage 10. Human Epithelial Kidney Cells (HEK-293) (ATCC Cat# CRL-1573) were maintained at 37°C with 5% CO2 in RPMI supplemented with 10% FBS, 10mM L-glutamine, 100 U/mL penicillin/100 mg/mL streptomycin (P/S), in the same way that HeLa Cells (ATCC Cat# CCL2), but in DMEM media. Aedes albopictus clone C6/36 HT cells (ATCC Cat# CRL-1660) were cultured at 34°C with 5% CO2 in L-15 media supplemented with 10% FBS and 1X P/S. Dengue virus Serotype 2 (DENV-2, New Guinea) was amplified in C6/36 HT cells grown in L-15 medium supplemented with 10% FBS, 100 U/mL P/S in T75 flasks at 70% confluence and incubated at 34°C [14]. The virus was titrated in BHK-21 hamster kidney cells (ATCC Cat# CCL-10) as described [14,62].

### 4.2. Generation of conditioned media

CMDV was obtained using the protocol described in [13]. Briefly, HMEC-1 cells at 75% confluence were infected with Dengue virus serotype 2 (DENV-2) obtained from viral amplification in C6/36 cells and titrated in BHK-21 cells as described [16]. HMEC-1 infection was carried out at a relative Multiplicity of Infection (MOI) of 5 for 2 hours. Subsequently, the cells were rinsed with Phosphate-Buffered Saline (PBS) and cultured in RPMI supplemented with 2% Fetal Bovine Serum (FBS) for 48 hours. Cell culture supernatants were collected and transferred to Petri dishes for complete virus inactivation using ultraviolet (UV) irradiation for 15 minutes. UV inactivation was confirmed by the absence of cytopathic effects in HMEC-1 cells for 6 days [16] and lack of PFU in BHK-21 cells. Inactivated supernatants were stored at −80°C for further use [14]. For experimental treatments, a ratio of 80:20 was used (80% CCM or CMDV and 20% fresh medium).

### 4.3. Fluorescence microscopy and image quantification

For the 48-hour assays, 1.5 × 10^5^ HMEC-1 cells were seeded into glass coverslips previously treated with 4% porcine gelatin and cultured for four days in 2% FBS + RPMI. Cells were then treated with CMDV in an 80:20 ratio [14], with conditioned medium from healthy cells (negative control), or with 5 ng/mL TGF-β1 (positive control). Cells were re-stimulated after 24 hours, then fixed after 48 h from the beginning of the assay. All conditions maintained a final concentration of 0.1% FBS in RPMI media.

For the 120-hour and imatinib assays, 4.0x10^4^ cells were seeded as above. After 24h, medium was switched to 0.1% FBS to favor the formation of cell-cell junctions and cells remained in these conditions for another 48h. Then, cells were treated with CMDV in the presence or absence of 6.25 µM imatinib (this concentration was determined experimentally as outlined in [14]) and fixed after 48h (120h total time).

After the cells were exposed to CMDV per 48 h or 120h, or to CMDV and then to imatinib per 48h or 120h, they were washed three times with 1 × Cytoskeletal Buffer with sucrose (CBS) [14], thereafter, they were fixed with 3.8% paraformaldehyde (PFA) in CBS for 20 minutes at 37°C. The cells were treated with NH_4_Cl (50 mM), permeabilized (0.02% Triton), and blocked (PBS + 5% FBS) for 1 hour at 37°C. Subsequently, the fixed cells were labeled at a 1:400 dilution with primary antibodies (mouse anti-Snail/VE-Cadherin /N-Cadherin or Occludin (OCLN)) for 1 hour at 37°C. After three thorough PBS washes, the cells were incubated with species-matched secondary antibodies coupled to AlexaFluor-594 or AlexaFluor488 as indicated, and Hoechst (1:5000) for 45 minutes at 37°C and finally mounted on slides using FluorSave mounting medium. For image acquisition, we used a Leica Thunder Tissue Analyzer (Leica Microsystems, GmbH) microscope equipped with a multi-LED using a 63x objective. Data processing and quantification were performed using ImageJ. To minimize observer bias, all image files were randomized and blinded before analysis. Data processing was performed using ImageJ version 1.54f, where the Integrated Density (IntDen) for both red and green channels was recorded. To maintain data quality, an outlier removal step was implemented using the Interquartile Range (IQR) method (values outside Q1-1.5\times IQR and Q3 + 1.5\times IQR were excluded). Final fluorescence intensity values were expressed as a percentage of expression relative to the total intensity to account for variations in cell density across treatments. Analysis was conducted using R software (version 4.3.0) with at least three biological replicates per condition. Given that the data did not meet the assumptions of normality (Shapiro-Wilk test) or homogeneity of variance (Bartlett’s test), non-parametric Kruskal-Wallis test was used, followed by a Dunn’s post-hoc test with Bonferroni correction. Multiple comparisons were controlled using Benjamini–Hochberg where relevant.

### 4.4. RNAseq-bulk sample generation and processing

HMEC-1 cells were seeded at 5 × 10^5^ per well (6-multiwell plate) in 10% RPMI at 37°C with 5% CO_2_ overnight to promote adherence. The cells were treated with 5 ng/mL TGF-β1 (positive control), CMDV, or conditioned medium from untreated cells (control) at a final concentration of 0.1% FBS. Medium was changed every 24h, and cells were collected for RNA extraction at the indicated time points. Three technical replicates and three biological replicates were performed per assay. Total RNA extraction was performed using the easyRNA kit (QiaGen) according to the manufacturer’s protocol. RNA quantity was assessed using Nanodrop and Qubit. Additionally, the quality was assessed according to the RNA Integrity Number (RIN) using Tapestation 4150 (Agilent) to select samples with a RIN ≥ 7 for library preparation. Library construction was carried out using MGI technology (MGISP-100 system, MGI. Shenzhen, China) in accordance with the manufacturer’s instructions and paired-end sequencing (2 × 100 bp). High-throughput sequencing was performed using the DNBSEQ-G50RS system (MGI, Shenzhen, China) with an SE 100 flowcell, spanning 120 cycles. The sequencing was performed at the National Genomics Laboratory of Colombia. Sequencing was conducted to achieve a depth of approximately 40 million reads per sample.

### 4.5. Sequencing data processed and Differential expressed genes (DEGs) analysis

Raw sequencing reads were processed to remove adapters and low-quality bases using Trimmomatic v0.39 [63] with the settings LEADING:3, TRAILING:3, MINLEN:50 CROP: 92,HEADCROP:8, SLIDINGWINDOW:4:20. After trimming (Trimmomatic) and read-level QC (FastQC), we assessed sample-level QC using variance-stabilized counts (DESeq2) and performed principal component analysis and hierarchical clustering to confirm replicate concordance and to identify potential outliers. No samples met exclusion criteria based on low library complexity or abnormal mapping/QC metrics. Quality control of the trimmed reads was performed with FastQC v0.11.9 [64], and clean reads were aligned to the reference genome GRCh38 (GCA_000001405.15 GCF_000001405.26) using STAR v2.7.10a [65] with the parameters --outFilterType BySJout --outFilterMultimapNmax 20 --alignSJoverhangMin 8 --alignSJDBoverhangMin 1 --outFilterMismatchNmax 999 --outFilterMismatchNoverLmax 0.04 --alignIntronMin 20 --alignIntronMax 1000000 --seedSearchStartLmax 30. Gene-level read counts were quantified using featureCounts v2.0.1 [66] with annotation files from GENCODE v39 [67]. Differential expression was performed with DESeq2 v1.36.0 [68]; p-values were adjusted using the Benjamini–Hochberg procedure and genes were considered significant at FDR < 0.05 with |log2FC| ≥ 1. All downstream RNA-seq-derived gene sets used for modeling were restricted to this FDR-controlled list. Data visualization, including heatmaps and volcano plots, was performed using the R package ggplot2 v3.3.6.

### 4.6. Gene ontology

Results from DEGs were used for functional enrichment analysis utilizing the Enrichr web tool version 2016 update [69]. We used Gene Ontology (GO) annotation to identify biological process enrichment, molecular functions, and cellular components associated with these DEGs. Only up regulated genes with p < 0.05 were used, and the accepted match are those with a adjusted p-value<0.01 and the combined score was high because the odds ratio varied for each suggested biological process, pathway or function. Additionally, the DEGs from CMDV at 48h and 120h were used to identify potential pharmacological targets through The Drug Gene Interaction Database. The results were filtered based on the interaction score (>4) and FDA approval. Additionally, the DEGs from CMDV at 48h and 120h were used to perform an interactome analysis in the StringDB database. The following parameters were applied: H. sapiens as the biological model, a high confidence level of 0.9, 1% FDR stringency, and clustering using MCL with an inflation factor of 3, which were classified in three biological phenomena: immune/inflammatory response, cellular migration and cellular plasticity/differentiation. The non-interacting nodes were excluded. The merge network between the two cellular responses was isolated and analyzed in cytoscape v 3.10.1, using Dynet Analyzer plugin to detect overlaps.

### 4.7. RT-qPCR

Expression levels of SNA1, TWIST,1 and VIM were assessed. ACTB (beta-actin gene) was used as housekeeping control. Primer sequences used are shown in S10 Table. cDNA was synthesized using M-MLV reverse transcriptase (Promega) from 500 ng of starting RNA. The reaction mix included random primers at a concentration of 125 ng/µL, 10 mM dNTP Mix, 50 mM Mg2 + 5x First-Strand Buffer, 100 nM DTT, RNase Out at 40 U/µL and MilliQ water, with a final volume of 20 µL. The thermal profile was as follows: 65°C for 5 minutes (incubation), 37°C for 2 minutes, 25°C for 10 minutes, 7°C for 50 minutes, and 70°C for 15 minutes (enzyme inactivation).

qPCR was conducted using SsoFastTM EvaGreen® Supermix (Biorad). Samples contained 400 nM primers, 1 µL of cDNA in a final volume of 9 µL. Amplification was performed in a CFX96TM TOUCH REAL-TIME PCR (Biorad) with the following thermal profile: 95°C for 30 seconds, followed by 40 cycles of 95°C for 5 seconds, 60°C for 5 seconds, and a gradual increase from 65°C to 95°C at a rate of 0.5°C every 5 seconds in an optical 96 multiwell plate. All reactions were performed in triplicate, and the mean Ct (Cycle threshold – fluorescent cycle threshold) was calculated for each sample.

mRNA differential expression was calculated using the 2 − ΔΔCt (Livak method), where ΔCt represents the difference between the Ct of the gene of interest and that of the housekeeping control ACTB). ΔΔCt is the comparison between the treated (CMDV and TGF- β1) samples and the controls (C-). The results were expressed as Log2 (LogFC). An increase in expression is indicated when LogFC > 1 and a decrease when LogFC < −1, following the methodology described by Coebergh Van Den Braak [70]. Differential expression, statistical analysis and graphics were performed in RStudio, with the packages tidyr v.1.31, dplyr v.1.14, qpcR v.1.4−1, stringr v.1.5.1, purrr v.1.0.2 and qPCRtools v.1.0.1. Statistical tests were carried out after assessing distributional assumptions (Shapiro–Wilk for normality; Levene’s test for homoscedasticity). Because these assumptions were not met, we used non-parametric tests (Mann–Whitney or Kruskal–Wallis with Dunn’s post hoc). Multiple comparisons were controlled using Benjamini–Hochberg where relevant.

### 4.8. Non-directed asynchronous boolean network

A subnetwork was obtained from the interactomes using the CMDVs DEGs created in StringDB to model a non-directed network. Therefore, bibliographic data was compiled to infer the node state where it was absent at one point and to understand its role in the biological processes and the model. For this reason, some interactions (edges) were included in the network to enhance its dynamism and to clarify the biological results.

The Boolean rules were formalized using Boolean algebra and were transcribed into a format suitable for use in the SPIDDOR v.1 RStudio packag. The number of edges and nodes, as well as centralities, the clustering coefficient, communities (Supplementary Information, Table B), and network metrics, were determined using the igraph v.2.1.2 package in RStudio v. 2024.12.0 (R v.4.3.0). To achieve the iterations necessary to activate the switch that changes between the 48-hour and 120-hour periods produced by the CMDV, a modified first-order decay model (1) was used [71], to find a solution to n (2).


$$\begin{array}{c} \frac{d(FN\text{1})}{dt}={FN\text{1}}_{max}-n\times {FN\text{1}}_{rate} \end{array}$$ (1)

with the condition that FN1 ≥ FN1_min_. FN1 represents the amount of FN1 at a given iteration, FN1_*max*_ is the maximum amount of FN1, n is the number of iterations and FN1_rate_ is the degradation rate of FN1

The equation (1) can be solved as follows:


$$\begin{array}{c} n=\frac{{FN\text{1}}_{max}-{FN\text{1}}_{min\;}}{{FN\text{1}}_{rate}} \end{array}$$ (2)

which is formulated considering the role of FN1 in the system and the biological process. The minimum and maximum values were arbitrary (0.1 and 1 (100%), respectively). Meanwhile, the reported degradation rate taken was 4.8% per 1h (it was taken as 0.04) which is the reported for normal catalysis [82]. The equation ignores the recovery rate. The advanced Boolean networks used in SPIDDOR defined the thresholds and modulators as described in S11 Table. The parameters to perform the average simulations, as well as to find the synchronous and asynchronous attractors and some interesting punctual perturbations such as knockouts or overexpression, were 100 iterations with 25000 repetitions in an asynchronous way. Meanwhile, to identify all the attractors, 6 CPUs were used with startStates set to 1000. The perturbation analysis evaluated the effects of single-node knockouts and single-node overexpression on the network. Sensitivity was disregarded due to its impact on the results. The model was exported to SBML (https://github.com/bioweaver/CMDV_nondirectModel_v.1.0). We deposited it in GitHub (https://github.com/bioweaver/CMDV_nondirectModel_v.1.0). Model visualization was created using ggplot v.3.5.0 packages.

### 4.9. Non-directed asynchronous boolean network

For the microscopy assays, data normality was assessed using the Shapiro-Wilk test and homogeneity was evaluated using the Bartlett test. Depending on the data distribution, one-way analysis of variance (ANOVA) was performed for normally distributed data, while the Kruskal-Wallis test was used for non-normally distributed data. Post-hoc analyses were conducted using Bonferroni in Dunnett tests or Tukey tests as appropriate. For the qPCR validation data, T test or Wilcoxon test were performed depending on the data distribution. Additionally, the mean and standard error of the mean (SEM) were calculated. For the non-directed network, the mean and standard deviation were calculated for the simulations and perturbation analyses performed.

#### 4.9.1. Node selection and edge support.

The nodes included in our model were prioritized from the differentially expressed genes (DEGs) identified at 48 and 120 hours post-exposure to CMDV. The selection and initial interaction mapping were performed using Cytoscape and STRING-db, with the latter providing the primary experimental and bibliographic support for each initial edge. While most nodes originate directly from our DEGs, some nodes presented “unknown” or inconsistent states at specific time points (e.g., at 48h vs. 120h). In these cases, we performed an extensive bibliographic search to determine the most probable biological state, ensuring the model remained consistent with the observed cellular response. Additionally, we manually verified interactions not initially detected by STRING-db or Cytoscape through literature review. These suggested interactions, which refine the molecular mechanisms affected by CMDV, are explicitly highlighted as predictions that require future experimental validation to increase the model’s robustness.

#### 4.9.2. Biological justification for the asynchronous model.

We selected a non-directed asynchronous paradigm because it more accurately reflects real biological scenarios compared to synchronous models. In a biological system, it is extremely rare for all molecular events to occur simultaneously. Asynchronous modeling assumes that variables are updated one at a time, capturing the stochastic nature of cellular processes. This is particularly relevant for describing multi-stability—the simultaneous presence of multiple state-space attractors (e.g., epithelial vs. mesenchymal states)—which describes how a single viral stimulus can lead to different cellular fates. Then, biological functions operate on vastly different timescales (e.g., fast protein-protein interactions vs. slower protein synthesis). Asynchronous and stochastic updating help mitigate the temporal distortions of synchronous models, allowing the representation of hierarchical dependencies, such as the requirement for a protein to be synthesized before it can be phosphorylated. In this models “complex attractors” emerge as overlapping loops in the state graph. This complexity is better suited for capturing system-wide biological behavior and cellular plasticity, as the trajectory of these graphs defines stable states (attractors) that represent biological equilibrium under specific conditions [72,73].

## Supporting information

S1 Fig Effect of exposure to imatinib for 48h in the expression of expression of selected endothelial and mesenchymal markers in endothelial cells in response to CMDV. Cells were treated with imatinib (10 µM) for 48h after 120h exposure to CMDV. Representative images of HMEC-1 cells treated as indicated and stained for DNA (DAPI) and N-Cadherin (A), VE-Cadherin (B) and occludin and Snail (C). In overlays, red is N-cadherin, VE-cadherin or occludin as indicated, and green is Snail; blue represents DAPI staining. TGF-β1 was used as a positive control. Scale bar = 20 µm. Under the microphotographs, quantitative analysis is shown. Data are presented as mean ± SEM from >100 fields examined in three independent experiments. See Material and Methods for additional details. Statistical tests are indicated, and significance is as follows: *p < 0.05, **p < 0.01, ***p < 0.001, ****p < 0.0001.
(PNG)

S2 Fig Effect of exposure to imatinib for 120h in the expression of selected endothelial and mesenchymal markers in endothelial cells in response to CMDV. Cells were treated with imatinib (10 µM) for 120h after 120h exposure to CMDV. Representative images of HMEC-1 cells treated as indicated and stained for DNA (DAPI) and N-Cadherin (A), VE-Cadherin (B) and occludin and Snail (C). In overlays, red is N-cadherin, VE-cadherin or occludin as indicated, and green is Snail; blue represents DAPI staining. TGF-β1 was used as a positive control. Scale bar = 20 µm. Under the microphotographs, quantitative analysis of images as in top. See Material and Methods for details. Data are presented as mean ± SEM from >100 fields examined in three independent experiments. Statistical tests are indicated, and significance is as follows: *p < 0.05, **p < 0.01, ***p < 0.001, ****p < 0.0001.
(PNG)

S3 Fig Heat maps of DEGs in HMEC-1 cells treated with CMDV for 48h. (PNG)

S4 Fig Heat maps of DEGs in HMEC-1 cells treated with CMDV for 120h. (PNG)

S5 Fig Heat maps of DEGs in HMEC-1 cells treated with TGF-β1 for 48h. (PNG)

S6 Fig Heat maps of DEGs in HMEC-1 cells treated with TGF-β1 for 120h. (PNG)

S7 Fig Gene enrichment analysis of HMEC-1 cells treated with CMDV for 48h. Only data points with p-values < 0.05 are shown. BP, Biological Processes; MF, Molecular Functions.
(PNG)

S8 Fig Gene enrichment analysis of HMEC-1 cells treated with TGF- β1 for 48h. (PNG)

S9 Fig Gene enrichment analysis of HMEC-1 cells treated with CMDV for 120h. (PNG)

S10 Fig Gene enrichment analysis of HMEC-1 cells treated with TGF- β1 for 120h. (PNG)

S11 Fig Interactome of HMEC-1 cells treated with CMDV for 48h. (PNG)

S12 Fig Interactome of HMEC-1 cells treated with CMDV for 120h. Dashed red box denotes clusters involved in cell cycle and division (clusters 1 and 2). Blue oval highlights cytokine signaling (cluster 7) and neighbors involved in ECM remodeling and cell migration (clusters 13, 15 and 29).
(PNG)

S13 Fig Merged network using DEGs from CDMV at 48h and 120h. The cellular response between the two times is not isolated and are biologically involved. The networks were grouped using the MCL clustering algorithm, representing functional groups and were classified in immune/inflammatory response (yellow clusters), migration (purple clusters) and cellular plasticity/differentiation (blue cluster). The red nodes and edges are from the cellular response at 48 h to CMDV, the green ones are from 120h, and the whites/grey are the overlap nodes and edges between the two times. Blue denotes the central nodes in the network.
(PNG)

S14 Fig Simulations of perturbation scenarios in response to CMDV in the non-directed network. Modulation of gene modulation in response to (A) PTGS2 knockout; (B) IL6 knockout; (C) FGF2 overexpression+FN1 knockout. For the analysis of single-gene perturbations (A-C), 25000 repetitions were performed. Pale magenta lines represent the state of each node in untreated conditions (as in Figure 6A).
(PNG)

S15 Fig Sensitivity over perturbation analysis. (A) Sensitivity over Perturbation in single node knockouts. (B) Sensitivity over Perturbation in single node overexpression.
(PNG)

S1 Table Potential Pharmacological Targets detected in CMDV DEGs. (XLSX)

S2 Table Gene status at 48h and 120h for CMDV model (includes additional references). (DOCX)

S3 Table A. Centrality analysis; B. Network metrics. (DOCX)

S4 Table CMDV network Simulation Averages. (XLSX)

S5 Table Attractors Analysis. (XLSX)

S6 Table PTGS2 knockout scenario in CMDV network. (XLSX)

S7 Table IL6 knockout scenario in CMDV network. (XLSX)

S8 Table Triple Knockout simulation AVG in CMDV network. (XLSX)

S9 Table FGF2 overexpression and FN1 knockout scenario AVG in CMDV network. (XLSX)

S10 Table qPCR Primer sequences. (DOCX)

S11 Table Thresholds and modulators in the advanced Boolean rules to describe the effect of CMDV on endothelial cells. (DOCX)

S1 File Extended results. (DOCX)
