# Supplementary figures and images for: Molecular analysis and computational modeling reveal temporally separable responses triggered by DENV-induced soluble factors in endothelial cells
Source: PLoS One. 2026 Jul 31;21(7):e0354877. doi: 10.1371/journal.pone.0354877 (PMC13426972; doi:10.1371/journal.pone.0354877)

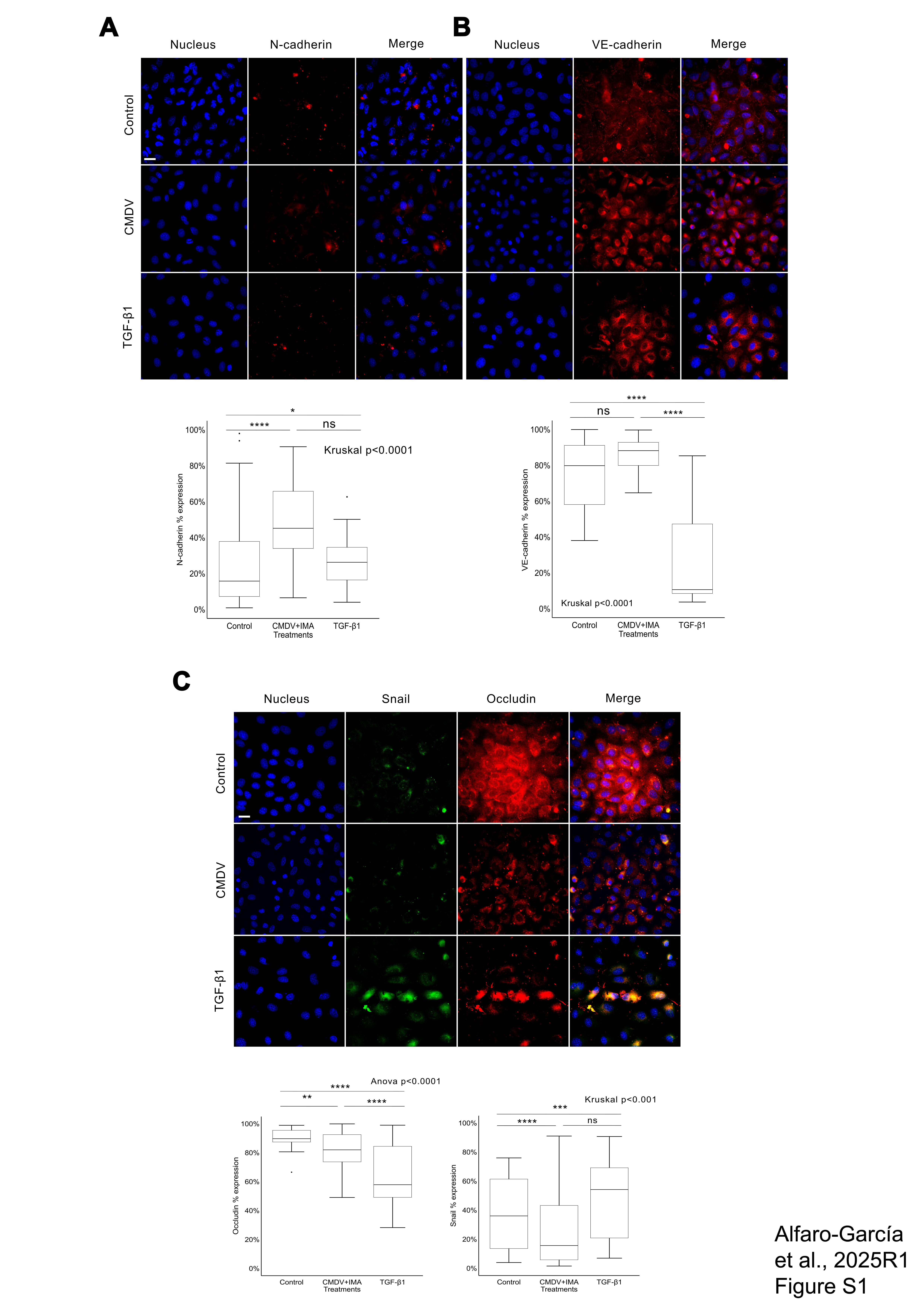

Supplement: S1 Fig — Cells were treated with imatinib (10 µM) for 48h after 120h exposure to CMDV. Representative images of HMEC-1 cells treated as indicated and stained for DNA (DAPI) and N-Cadherin (A), VE-Cadherin (B) and occludin and Snail (C). In overlays, red is N-cadherin, VE-cadherin or occludin as indicated, and green is Snail; blue represents DAPI staining. TGF-β1 was used as a positive control. Scale bar = 20 µm. Under the microphotographs, quantitative analysis is shown. Data are presented as mean ± SEM from >100 fields examined in three independent experiments. See Material and Methods for additional details. Statistical tests are indicated, and significance is as follows: *p < 0.05, **p < 0.01, ***p < 0.001, ****p < 0.0001. (PNG) [file pone.0354877.s001.png]

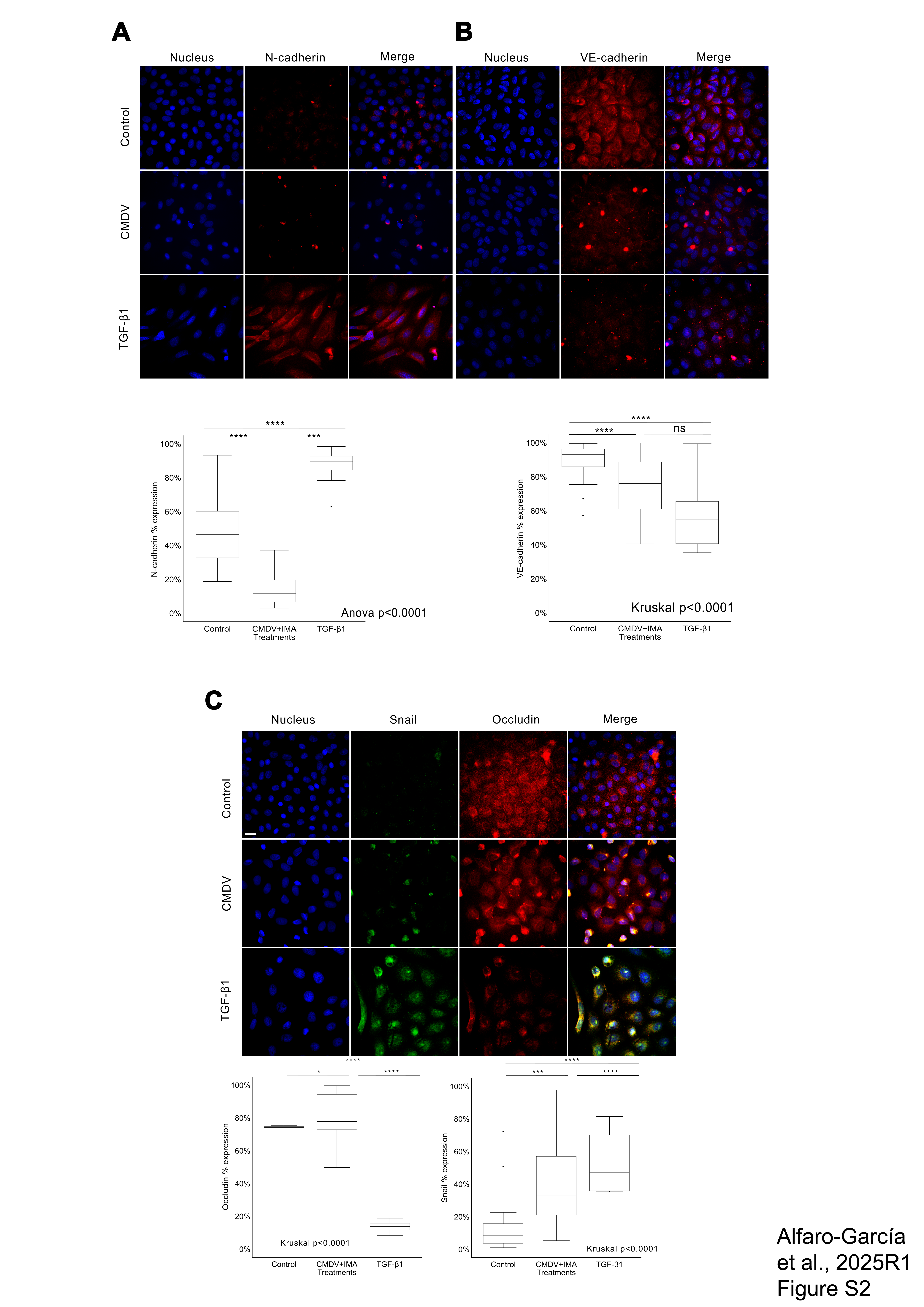

Supplement: S2 Fig — Cells were treated with imatinib (10 µM) for 120h after 120h exposure to CMDV. Representative images of HMEC-1 cells treated as indicated and stained for DNA (DAPI) and N-Cadherin (A), VE-Cadherin (B) and occludin and Snail (C). In overlays, red is N-cadherin, VE-cadherin or occludin as indicated, and green is Snail; blue represents DAPI staining. TGF-β1 was used as a positive control. Scale bar = 20 µm. Under the microphotographs, quantitative analysis of images as in top. See Material and Methods for details. Data are presented as mean ± SEM from >100 fields examined in three independent experiments. Statistical tests are indicated, and significance is as follows: *p < 0.05, **p < 0.01, ***p < 0.001, ****p < 0.0001. (PNG) [file pone.0354877.s002.png]

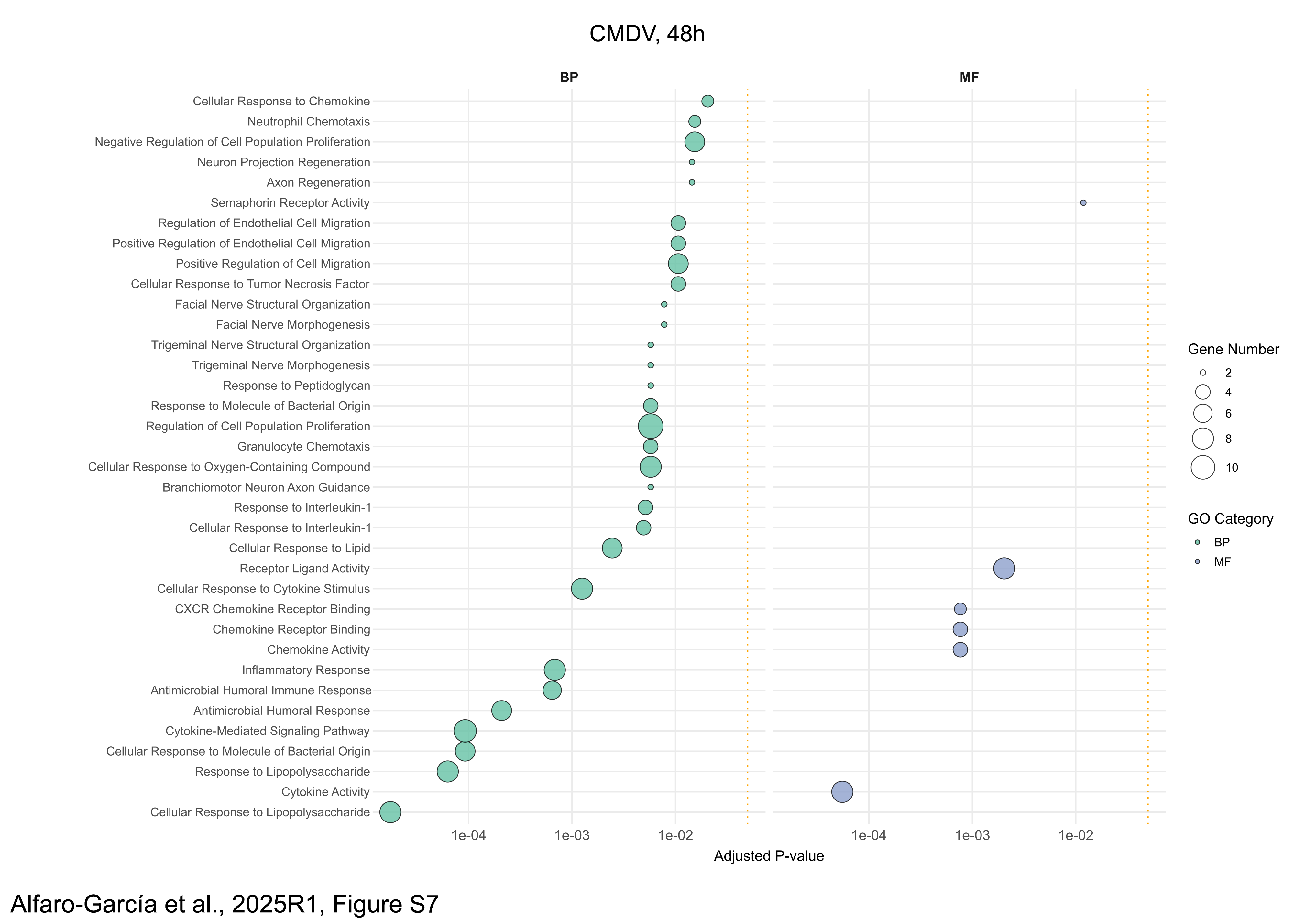

Supplement: S7 Fig — Only data points with p-values < 0.05 are shown. BP, Biological Processes; MF, Molecular Functions. (PNG) [file pone.0354877.s007.png]

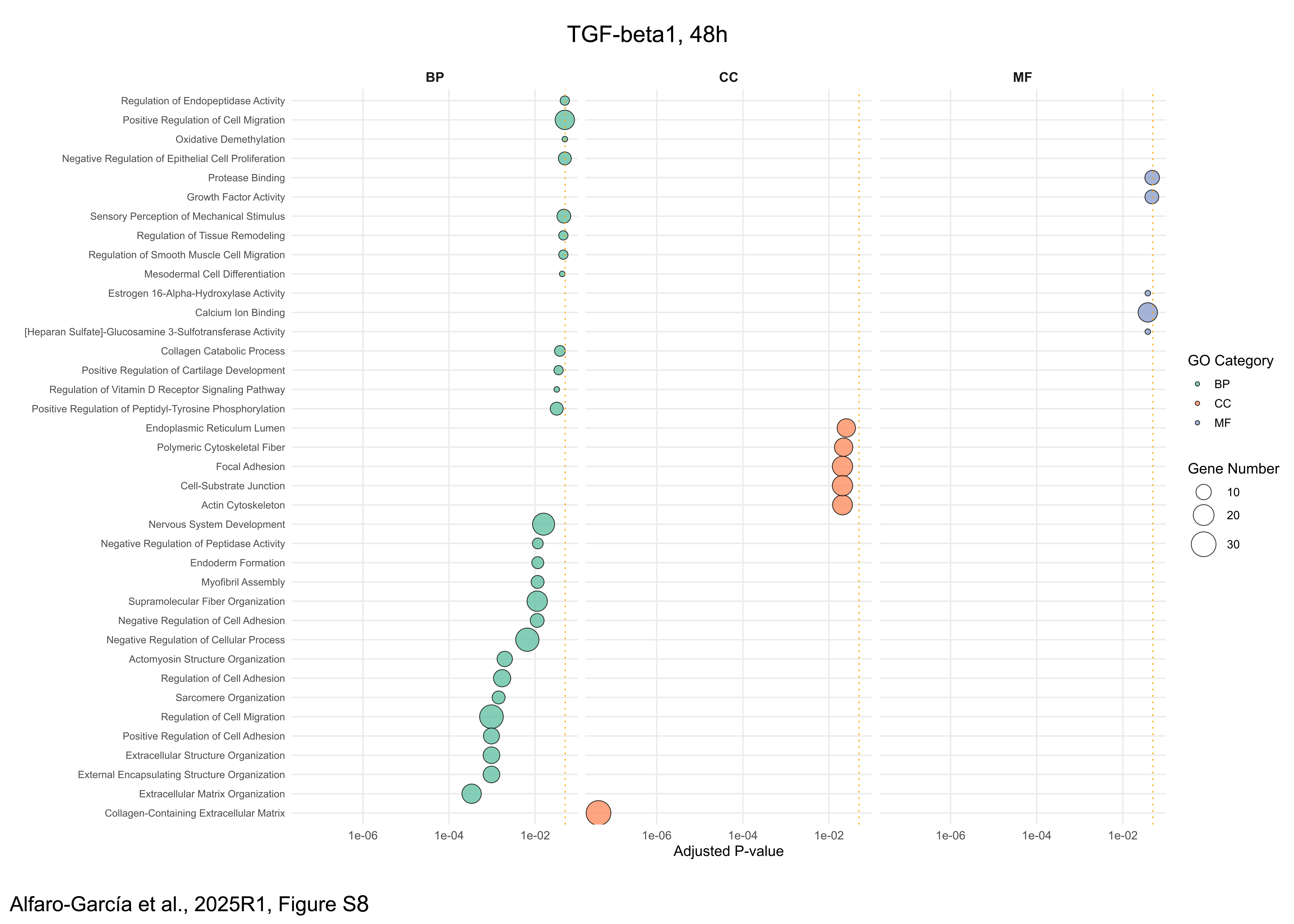

Supplement: S8 Fig — (PNG) [file pone.0354877.s008.png]

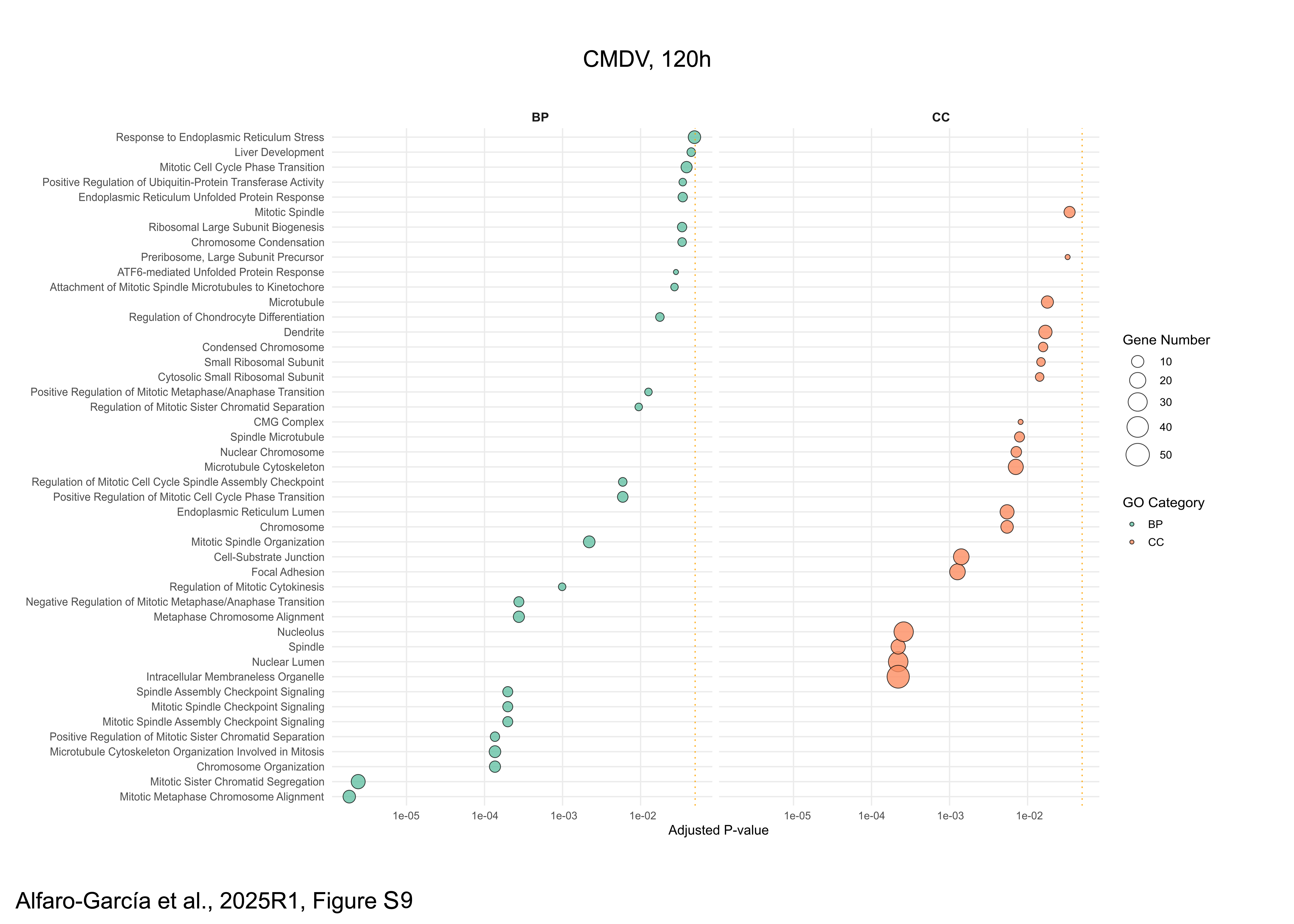

Supplement: S9 Fig — (PNG) [file pone.0354877.s009.png]

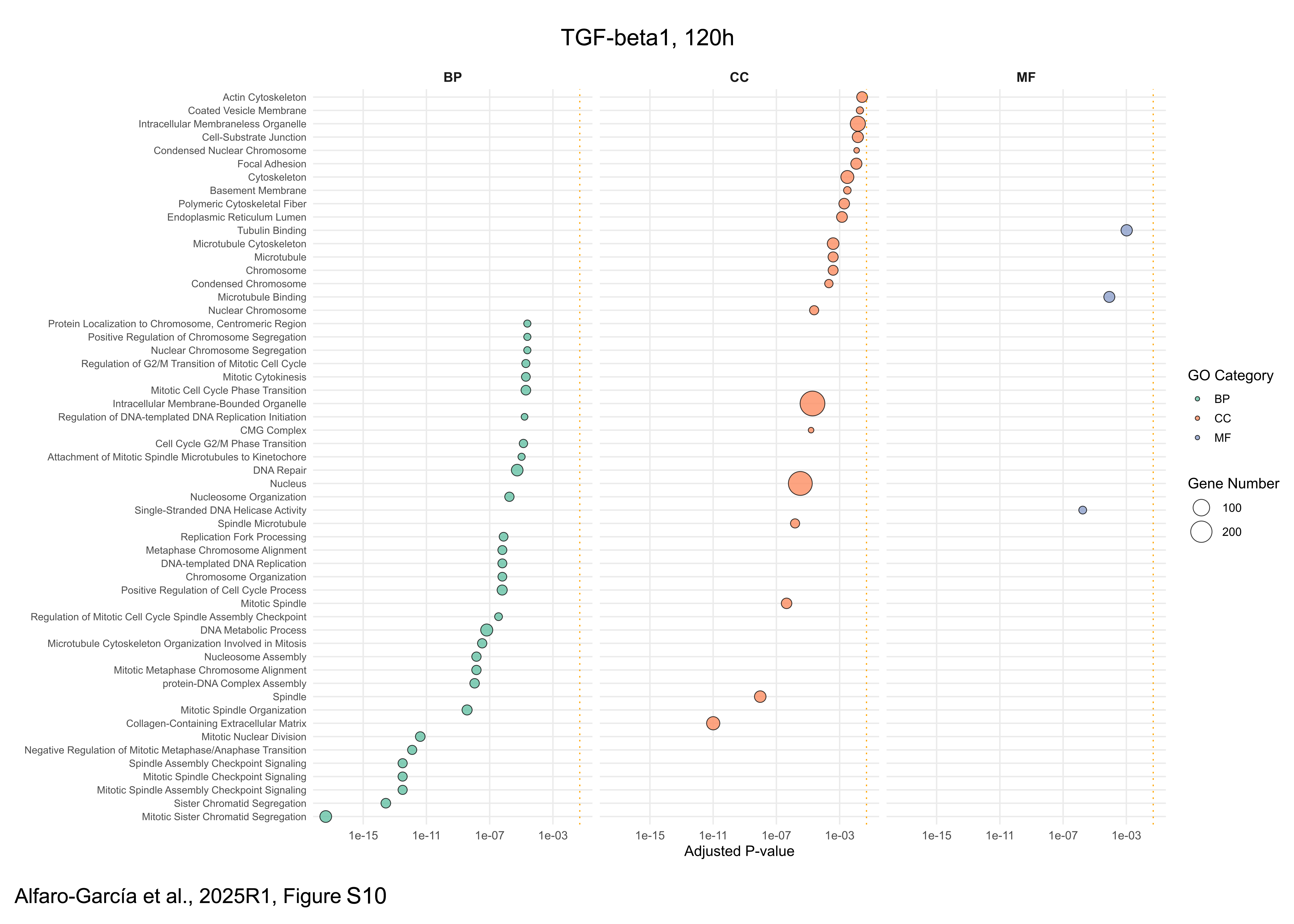

Supplement: S10 Fig — (PNG) [file pone.0354877.s010.png]

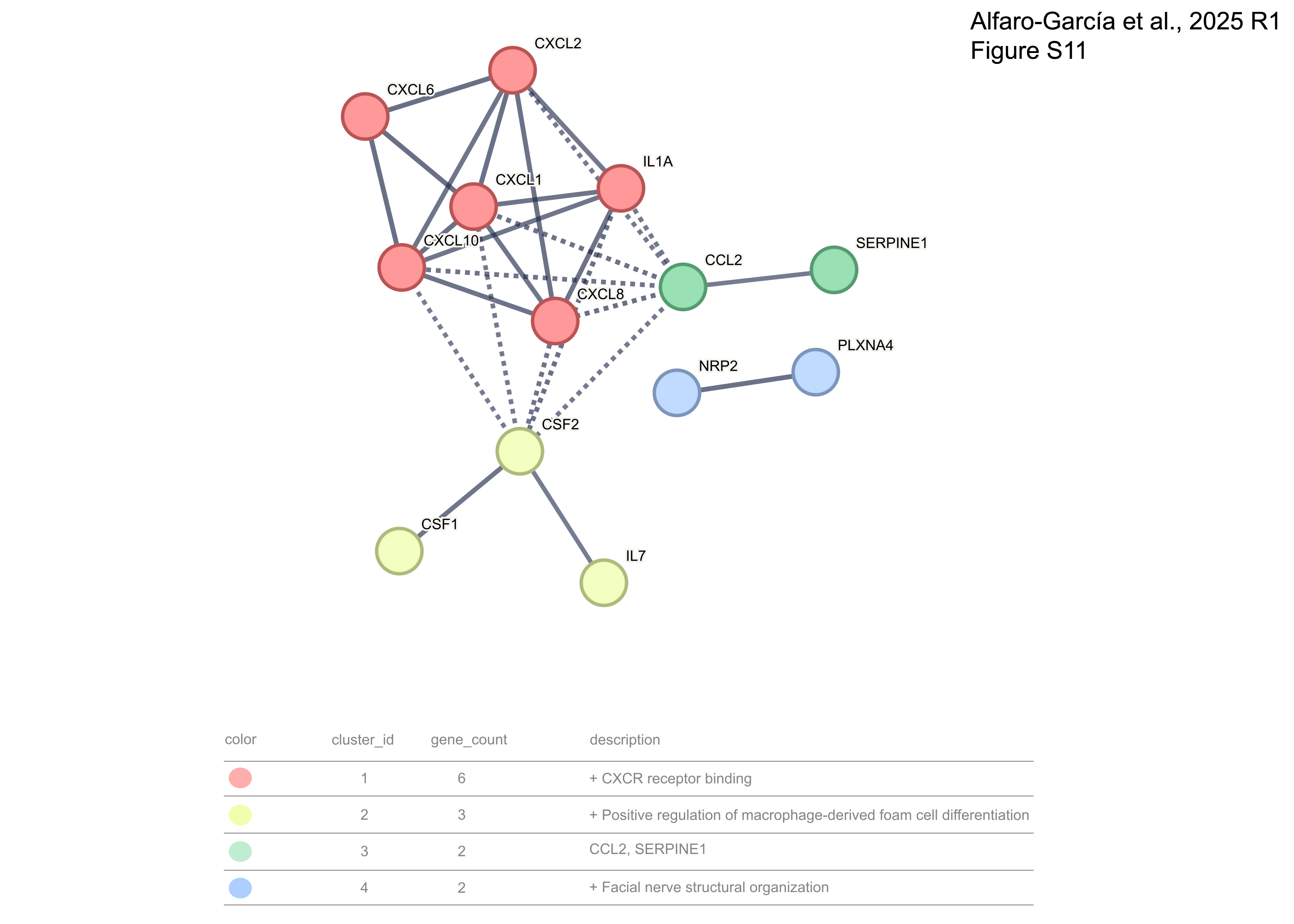

Supplement: S11 Fig — (PNG) [file pone.0354877.s011.png]

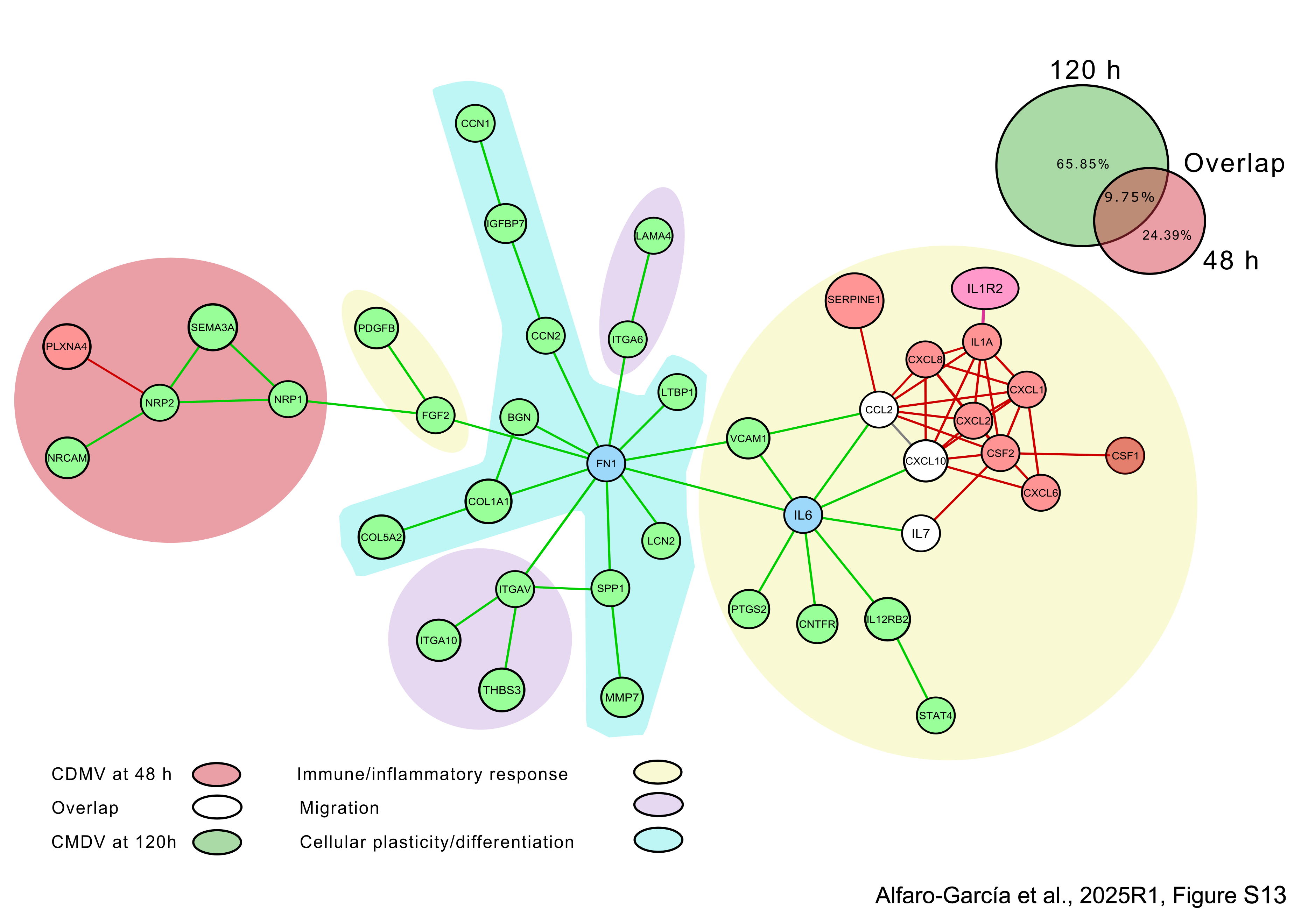

Supplement: S13 Fig — The cellular response between the two times is not isolated and are biologically involved. The networks were grouped using the MCL clustering algorithm, representing functional groups and were classified in immune/inflammatory response (yellow clusters), migration (purple clusters) and cellular plasticity/differentiation (blue cluster). The red nodes and edges are from the cellular response at 48 h to CMDV, the green ones are from 120h, and the whites/grey are the overlap nodes and edges between the two times. Blue denotes the central nodes in the network. (PNG) [file pone.0354877.s013.png]

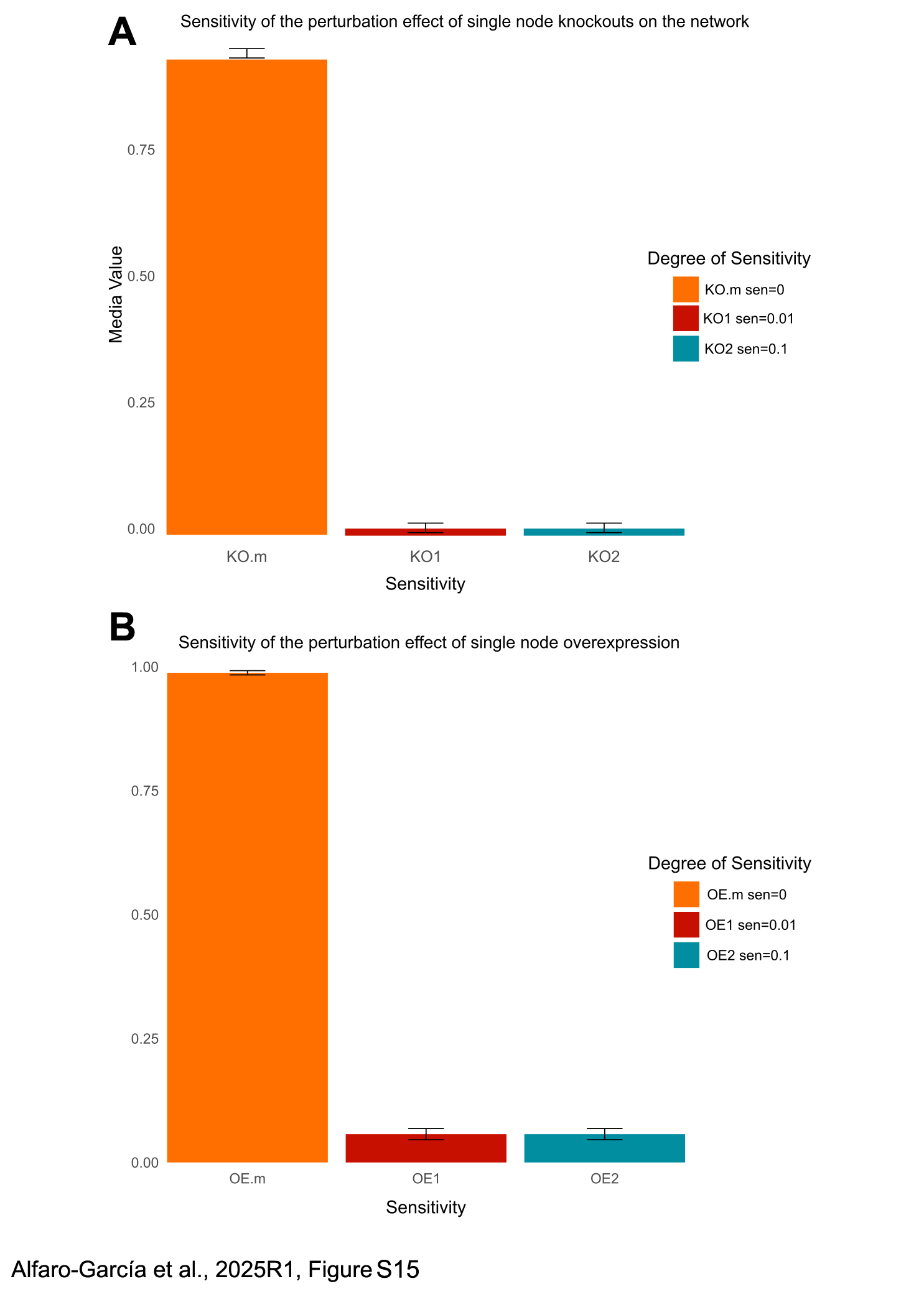

Supplement: S15 Fig — (A) Sensitivity over Perturbation in single node knockouts. (B) Sensitivity over Perturbation in single node overexpression. (PNG) [file pone.0354877.s015.png]
