# Supplementary material for: Molecular analysis and computational modeling reveal temporally separable responses triggered by DENV-induced soluble factors in endothelial cells
Source: PLoS One. 2026 Jul 31;21(7):e0354877. doi: 10.1371/journal.pone.0354877 (PMC13426972; doi:10.1371/journal.pone.0354877)
Supplement: S2 Table — (DOCX) [file pone.0354877.s017.docx]

Alfaro-García et al., 2025, Supplementary Table 2

| **Supplementary Table 2. Gene status at 48h and 120h for CMDV model** | | | |
| --- | --- | --- | --- |
| **Gene** | **48h** | **120h** | **References** |
| STAT4 | **up** | up | (1,2) |
| IL12RB2 | **up** | down | (3) |
| CNTFR | up | down | (4) |
| IL6 | **up** | up | (5–7) |
| VCAM1 | **up** | down | (8,9) |
| IL7 | up | down | (10,11) |
| CXCL1 | up | **down** | (7,12) |
| CCL2 | up | down |  |
| SERPINE1 | down | **up** | (13) |
| CXCL10 | up | down | (7,14) |
| CXCL8 | up | **down** | (14–16) |
| IL1A | up | **down** | (17,18) |
| CXCL2 | up | **down** | (12,14) |
| PTGS2 | **up** | down | (19–21) |
| CXCL6 | up | **down** | (14,16) |
| IL1R2 | **up** | up | (22) |
| CSF2 | up | **down** | (23,24) |
| CSF1 | up | down |  |
| FGF2 | **up** | up | (25,26) |
| PDGFB | **up** | down | (27,28) |
| NRP1 | **up** | up | (29–37) |
| SEMA3A | **up** | up |  |
| NRP2 | up | down |  |
| NRCAM | **down** | up |  |
| PLXNA4 | up | **down** |  |
| FN1 | **up** | down | (38,39) |
| LCN2 | **up** | up | (40–42) |
| BGN | **up** | up | (43) |
| COL1A1 | **up** | down | (44,45) |
| COL5A2 | down | up | (46,47) |
| ITGA6 | down | up | (48,49) |
| LAMA4 | down | up |  |
| LTBP1 | **up** | up | (50,51) |
| ITGAV | **up** | down | (52,53) |
| THBS3 | **up** | down | (54) |
| ITGA10 | **up** | down | (55,56) |
| SPP1 | **up** | down | (57,58) |
| MMP7 | **up** | down | (59,60) |
| CCN2 | **up** | up | (61,62) |
| CCN1 | **up** | up | (63,64) |
| IGFBP7 | up | down | (65,66) |

Bold represents the assumed state, or trend, of the indicated gene after incubation of HMEC-1 cells with CMDV for 48h or 120h

**Supplementary bibliography**

1. Meng ZZ, Liu W, Xia Y, Yin HM, Zhang CY, Su D, et al. The pro-inflammatory signalling regulator Stat4 promotes vasculogenesis of great vessels derived from endothelial precursors. Nat Commun [Internet]. 2017;8:1–12. Available from: http://dx.doi.org/10.1038/ncomms14640

2. Nguyen HN, Noss EH, Mizoguchi F, Huppertz C, Wei KS, Watts GFM, et al. Autocrine Loop Involving IL-6 Family Member LIF, LIF Receptor, and STAT4 Drives Sustained Fibroblast Production of Inflammatory Mediators. Immunity [Internet]. 2017;46(2):220–32. Available from: http://dx.doi.org/10.1016/j.immuni.2017.01.004

3. Yao BB, Niu P, Surowy CS, Faltynek CR. Direct interaction of STAT4 with the IL-12 receptor. Arch Biochem Biophys. 1999;368(1):147–55.

4. Zhang L, Xiang Y, Cao C, Tan J, Li F, Yang X. Ciliary neurotrophic factor promotes the development of homocysteine-induced vascular endothelial injury through inflammation mediated by the JAK2/STAT3 signaling pathway. Exp Cell Res [Internet]. 2024;440(1):114103. Available from: https://www.sciencedirect.com/science/article/pii/S0014482724001940

5. Vachher H, Metgud T, Srikanth B. IL-6 Levels in Prediction of Severity of Dengue Fever. Indian J Pediatr. 2023;90(5):518.

6. Jovanović M, Vićovac L. Interleukin-6 Stimulates Cell Migration, Invasion and Integrin Expression in HTR-8/SVneo Cell Line. Placenta [Internet]. 2009;30(4):320–8. Available from: http://dx.doi.org/10.1016/j.placenta.2009.01.013

7. Jusof FF, Lim CK, Aziz FN, Soe HJ, Raju CS, Sekaran SD, et al. The Cytokines CXCL10 and CCL2 and the Kynurenine Metabolite Anthranilic Acid Accurately Predict Patients at Risk of Developing Dengue with Warning Signs. J Infect Dis [Internet]. 2022;226(11):1964–73. Available from: https://doi.org/10.1093/infdis/jiac273

8. Cook-Mills JM, Marchese ME, Abdala-Valencia H. Vascular cell adhesion molecule-1 expression and signaling during disease: Regulation by reactive oxygen species and antioxidants. Antioxidants Redox Signal. 2011;15(6):1607–38.

9. Pickett JR, Wu Y, Zacchi LF, Ta HT. Targeting endothelial vascular cell adhesion molecule-1 in atherosclerosis: drug discovery and development of vascular cell adhesion molecule-1-directed novel therapeutics. Cardiovasc Res [Internet]. 2023;119(13):2278–93. Available from: https://doi.org/10.1093/cvr/cvad130

10. Winer H, Rodrigues GOL, Hixon JA, Aiello FB, Hsu TC, Wachter BT, et al. IL-7: Comprehensive review. Cytokine [Internet]. 2022;160:156049. Available from: https://www.sciencedirect.com/science/article/pii/S1043466622002587

11. Ariel A, Hershkoviz R, Cahalon L, Williams DE, Akiyama SK, Yamada KM, et al. Induction of T cell adhesion to extracellular matrix or endothelial cell ligands by soluble or matrix-bound interleukin-7. Eur J Immunol. 1997;27(10):2562–70.

12. Boro M, Balaji KN. CXCL1 and CXCL2 Regulate NLRP3 Inflammasome Activation via G-Protein–Coupled Receptor CXCR2. J Immunol. 2017;199(5):1660–71.

13. Simone TM, Higgins CE, Czekay RP, Law BK, Higgins SP, Archambeault J, et al. SERPINE1: A Molecular Switch in the Proliferation-Migration Dichotomy in Wound-“Activated” Keratinocytes. Adv Wound Care. 2014;3(3):281–90.

14. Zhou C, Gao Y, Ding P, Wu T, Ji G. The role of CXCL family members in different diseases. Cell Death Discov. 2023;9(1):1–12.

15. Ji HZ, Chen L, Ren M, Li S, Liu TY, Chen HJ, et al. CXCL8 Promotes Endothelial-to-Mesenchymal Transition of Endothelial Cells and Protects Cells from Erastin-Induced Ferroptosis via CXCR2-Mediated Activation of the NF-κB Signaling Pathway. Pharmaceuticals. 2023;16(9).

16. Cambier S, Gouwy M, Proost P. The chemokines CXCL8 and CXCL12: molecular and functional properties, role in disease and efforts towards pharmacological intervention. Cell Mol Immunol. 2023;20(3):217–51.

17. Liu X, Zhang H, He S, Mu X, Hu G, Dong H. Endothelial-Derived Interleukin-1α Activates Innate Immunity by Promoting the Bactericidal Activity of Transendothelial Neutrophils. Front Cell Dev Biol. 2020;8(July):1–9.

18. Cavalli G, Colafrancesco S, Emmi G, Imazio M, Lopalco G, Maggio MC, et al. Interleukin 1α: a comprehensive review on the role of IL-1α in the pathogenesis and treatment of autoimmune and inflammatory diseases. Autoimmun Rev [Internet]. 2021;20(3):102763. Available from: https://doi.org/10.1016/j.autrev.2021.102763

19. Kulesza A, Paczek L, Burdzinska A. The Role of COX-2 and PGE2 in the Regulation of Immunomodulation and Other Functions of Mesenchymal Stromal Cells. Biomedicines. 2023;11(2).

20. Neil JR, Johnson KM, Nemenoff RA, Schiemann WP. Cox-2 inactivates Smad signaling and enhances EMT stimulated by TGF-β through a PGE2-dependent mechanisms. Carcinogenesis. 2008;29(11):2227–35.

21. Lin CK, Tseng CK, Wu YH, Liaw CC, Lin CY, Huang CH, et al. Cyclooxygenase-2 facilitates dengue virus replication and serves as a potential target for developing antiviral agents. Sci Rep. 2017;7(August 2016):1–15.

22. Peters VA, Joesting JJ, Freund GG. IL-1 receptor 2 (IL-1R2) and its role in immune regulation. Brain Behav Immun [Internet]. 2013;32:1–8. Available from: http://dx.doi.org/10.1016/j.bbi.2012.11.006

23. Park SR, Cho A, Kim JW, Lee HY, Hong IS. A Novel Endogenous Damage Signal, CSF-2, Activates Multiple Beneficial Functions of Adipose Tissue-Derived Mesenchymal Stem Cells. Mol Ther [Internet]. 2019;27(6):1087–100. Available from: https://doi.org/10.1016/j.ymthe.2019.03.010

24. Park KW, Kwon YW, Cho HJ, Shin JI, Kim YJ, Lee SE, et al. G-CSF exerts dual effects on endothelial cells-Opposing actions of direct eNOS induction versus indirect CRP elevation. J Mol Cell Cardiol [Internet]. 2008;45(5):670–8. Available from: http://dx.doi.org/10.1016/j.yjmcc.2008.07.002

25. Tan Y, Qiao Y, Chen Z, Liu J, Guo Y, Tran T, et al. FGF2, an Immunomodulatory Factor in Asthma and Chronic Obstructive Pulmonary Disease (COPD). Front Cell Dev Biol. 2020;8(April):1–12.

26. Piera-Velazquez S, Jimenez SA. Endothelial to mesenchymal transition: Role in physiology and in the pathogenesis of human diseases. Physiol Rev. 2019;99(2):1281–324.

27. Eng E, Ballermann BJ. Diminished NF-κB activation and PDGF-B expression in glomerular endothelial cells subjected to chronic shear stress. Microvasc Res. 2003;65(3):137–44.

28. D’Amore PA, Sakurai MK. Angiogenesis, Angiogenic Growth Factors and Development Factors. Encycl Respir Med Vol 1-4. 2006;1–4:V1-110-V1-115.

29. Sharma S, Ehrlich M, Zhang M, Blobe GC, Henis YI. NRP1 interacts with endoglin and VEGFR2 to modulate VEGF signaling and endothelial cell sprouting. Commun Biol. 2024;7(1):1–15.

30. Issitt T, Bosseboeuf E, De Winter N, Dufton N, Gestri G, Senatore V, et al. Neuropilin-1 Controls Endothelial Homeostasis by Regulating Mitochondrial Function and Iron-Dependent Oxidative Stress. iScience [Internet]. 2019;11:205–23. Available from: https://doi.org/10.1016/j.isci.2018.12.005

31. Chikh A, Raimondi C. Endothelial Neuropilin-1: a multifaced signal transducer with an emerging role in inflammation and atherosclerosis beyond angiogenesis. Biochem Soc Trans. 2024;52(1):137–50.

32. Tokudome T, Otani K, Mao Y, Jensen LJ, Arai Y, Miyazaki T, et al. Endothelial Natriuretic Peptide Receptor 1 Play Crucial Role for Acute and Chronic Blood Pressure Regulation by Atrial Natriuretic Peptide. Hypertension. 2022;79(7):1409–22.

33. Sogawa-Fujiwara C, Fujiwara Y, Hanagata A, Yang Q, Mihara T, Kaji N, et al. Npr2 mutant mice show vasodilation and undeveloped adipocytes in mesentery. BMC Res Notes [Internet]. 2021;14(1):1–7. Available from: https://doi.org/10.1186/s13104-021-05853-9

34. Špiranec K, Chen W, Werner F, Nikolaev VO, Naruke T, Werner FKA, et al. Endothelial C-type natriuretic peptide acts on pericytes to regulate microcirculatory flow and blood pressure. Circulation. 2018;138(5):494–508.

35. Acevedo LM, Barillas S, Weis SM, Göthert JR, Cheresh DA. Semaphorin 3A suppresses VEGF-mediated angiogenesis yet acts as a vascular permeability factor. Blood. 2008;111(5):2674–80.

36. Eberhard D, Balkenhol S, Köster A, Follert P, Upschulte E, Ostermann P, et al. Semaphorin-3A regulates liver sinusoidal endothelial cell porosity and promotes hepatic steatosis. Nat Cardiovasc Res. 2024;3(6):734–53.

37. Reidy KJ, Villegas G, Teichman J, Veron D, Shen W, Jimenez J, et al. Semaphorin3a regulates endothelial cell number and podocyte differentiation during glomerular development. Development. 2009;136(23):3979–89.

38. Luo X, Jian W. Different roles of endothelial cell-derived fibronectin and plasma fibronectin in endothelial dysfunction. Turkish J Med Sci. 2023;53(6):1667–77.

39. Al-Yafeai Z, Yurdagul A, Peretik JM, Alfaidi M, Murphy PA, Orr AW. Endothelial FN (Fibronectin) deposition by α5β1 integrins drives atherogenic inflammation. Arterioscler Thromb Vasc Biol. 2018;38(11):2601–14.

40. Sivakumar K, Subbiah U. Computational analysis of non-synonymous SNPs in the human LCN2 gene. Egypt J Med Hum Genet [Internet]. 2024;25(1). Available from: https://doi.org/10.1186/s43042-024-00565-8

41. Guardado S, Ojeda-Juárez D, Kaul M, Nordgren TM. Comprehensive review of lipocalin 2-mediated effects in lung inflammation. Am J Physiol - Lung Cell Mol Physiol. 2021;321(4):L726–33.

42. Kim SL, Shin MW, Seo SY, Kim SW. Lipocalin 2 potentially contributes to tumorigenesis from colitis via IL-6/STAT3/NF-κB signaling pathway. Biosci Rep. 2022;42(5):1–14.

43. Gáspár R, Diószegi P, Nógrádi-Halmi D, Erdélyi-Furka B, Varga Z, Kahán Z, et al. The Proteoglycans Biglycan and Decorin Protect Cardiac Cells against Irradiation-Induced Cell Death by Inhibiting Apoptosis. Cells. 2024;13(10).

44. Zeltz C, Orgel J, Gullberg D. Molecular composition and function of integrin-based collagen glues - Introducing COLINBRIs. Biochim Biophys Acta - Gen Subj [Internet]. 2014;1840(8):2533–48. Available from: http://dx.doi.org/10.1016/j.bbagen.2013.12.022

45. Singh D, Rai V, K Agrawal D. Regulation of Collagen I and Collagen III in Tissue Injury and Regeneration. Cardiol Cardiovasc Med. 2023;07(01):5–16.

46. Chen M, Zhu X, Zhang L, Zhao D. COL5A2 is a prognostic-related biomarker and correlated with immune infiltrates in gastric cancer based on transcriptomics and single-cell RNA sequencing. BMC Med Genomics [Internet]. 2023;16(1):1–20. Available from: https://doi.org/10.1186/s12920-023-01659-9

47. Yin W, Zhu H, Tan J, Xin Z, Zhou Q, Cao Y, et al. Identification of collagen genes related to immune infiltration and epithelial-mesenchymal transition in glioma. Cancer Cell Int [Internet]. 2021;21(1):1–18. Available from: https://doi.org/10.1186/s12935-021-01982-0

48. Xu H, Pumiglia K, LaFlamme SE. Laminin-511 and α6 integrins regulate the expression of CXCR4 to promote endothelial morphogenesis. J Cell Sci. 2020;133(11).

49. Béguin EP, Janssen EFJ, Hoogenboezem M, Meijer AB, Hoogendijk AJ, van den Biggelaar M. Flow-induced Reorganization of Laminin-integrin Networks Within the Endothelial Basement Membrane Uncovered by Proteomics. Mol Cell Proteomics. 2020;19(7):1179–92.

50. Cai R, Wang P, Zhao X, Lu X, Deng R, Wang X, et al. LTBP1 promotes esophageal squamous cell carcinoma progression through epithelial-mesenchymal transition and cancer-associated fibroblasts transformation. J Transl Med [Internet]. 2020;18(1):1–13. Available from: https://doi.org/10.1186/s12967-020-02310-2

51. Klingberg F, Chau G, Walraven M, Boo S, Koehler A, Chow ML, et al. The fibronectin ED-A domain enhances recruitment of latent TGF-β-binding protein-1 to the fibroblast matrix. J Cell Sci. 2018;131(5):1–12.

52. Zhang C, Wu M, Zhang L, Shang L ru, Fang J hong. Fibrotic microenvironment promotes the metastatic seeding of tumor cells via activating the fibronectin 1/secreted phosphoprotein 1-integrin signaling. Oncotarget. 2016;7(29).

53. Xu D, Li T, Wang R, Mu R. Expression and Pathogenic Analysis of Integrin Family Genes in Systemic Sclerosis. Front Med. 2021;8(July).

54. Pan H, Lu X, Ye D, Feng Y, Wan J, Ye J. The molecular mechanism of thrombospondin family members in cardiovascular diseases. Front Cardiovasc Med. 2024;11(March):1–12.

55. Wolpe AG, Ruddiman CA, Hall PJ, Isakson BE. Polarized Proteins in Endothelium and Their Contribution to Function. J Vasc Res. 2021;58(2):65–91.

56. Lemma SA, Kuusisto M, Haapasaari KM, Sormunen R, Lehtinen T, Klaavuniemi T, et al. Integrin alpha 10, CD44, PTEN, cadherin-11 and lactoferrin expressions are potential biomarkers for selecting patients in need of central nervous system prophylaxis in diffuse large B-cell lymphoma. Carcinogenesis. 2017;38(8):812–20.

57. Agnihotri R, Crawford HC, Haro H, Matrisian LM, Havrda MC, Liaw L. Osteopontin, a Novel Substrate for Matrix Metalloproteinase-3 (Stromelysin-1) and Matrix Metalloproteinase-7 (Matrilysin). J Biol Chem [Internet]. 2001;276(30):28261–7. Available from: http://dx.doi.org/10.1074/jbc.M103608200

58. Zhao Y, Huang Z, Gao L, Ma H, Chang R. Osteopontin/SPP1: a potential mediator between immune cells and vascular calcification. Front Immunol. 2024;15(June):1–11.

59. Quintero-Fabián S, Arreola R, Becerril-Villanueva E, Torres-Romero JC, Arana-Argáez V, Lara-Riegos J, et al. Role of Matrix Metalloproteinases in Angiogenesis and Cancer. Front Oncol. 2019;9(December):1–21.

60. Ito TK, Ishii G, Saito S, Yano K, Hoshino A, Suzuki T, et al. Degradation of soluble VEGF receptor-1 by MMP-7 allows VEGF access to endothelial cells. Blood. 2009;113(10):2363–9.

61. Chaqour B. Caught between a “Rho” and a hard place: are CCN1/CYR61 and CCN2/CTGF the arbiters of microvascular stiffness? J Cell Commun Signal. 2020;14(1):21–9.

62. Mo FE. Shear-Regulated Extracellular Microenvironments and Endothelial Cell Surface Integrin Receptors Intertwine in Atherosclerosis. Front Cell Dev Biol. 2021;9(April).

63. Yu Y, Gao Y, Qin J, Kuang CY, Song MB, Yu SY, et al. CCN1 promotes the differentiation of endothelial progenitor cells and reendothelialization in the early phase after vascular injury. Basic Res Cardiol. 2010;105(6):713–24.

64. Hsu PL, Chen JS, Wang CY, Wu HL, Mo FE. Shear-Induced CCN1 Promotes Atheroprone Endothelial Phenotypes and Atherosclerosis. Circulation. 2019;139(25):2877–91.

65. Surolia R, Zmijewski JW. IGFBP7: When Endothelial gCap-ing Goes Wrong in Acute Lung Injury. Am J Respir Cell Mol Biol. 2024;71(1):21–2.

66. He R, Feng B, Zhang Y, Li Y, Wang D, Yu L. IGFBP7 promotes endothelial cell repair in the recovery phase of acute lung injury. Clin Sci. 2024;138(13):797–815.
