# Supplementary material for: Molecular analysis and computational modeling reveal temporally separable responses triggered by DENV-induced soluble factors in endothelial cells
Source: PLoS One. 2026 Jul 31;21(7):e0354877. doi: 10.1371/journal.pone.0354877 (PMC13426972; doi:10.1371/journal.pone.0354877)
Supplement: S3 Table — (DOCX) [file pone.0354877.s018.docx]

| **Supplementary Table 3A. Centrality analysis** | | | | | |
| --- | --- | --- | --- | --- | --- |
| **Node** | **Degree** | **Betweenness** | | **Closeness** | **Eigenvector** |
| STAT4 | 5 | 0.5 | | 0.00892857 | 0.19954602 |
| IL6 | 22 | 312.779131 | | 0.01333333 | 1 |
| FN1 | 12 | 290.807504 | | 0.01315789 | 0.32518526 |
| IL1A | 11 | 13.799026 | | 0.01052632 | 0.80699843 |
| PTGS2 | 5 | 10.3804113 | | 0.01020408 | 0.33312293 |
| CXCL1 | 11 | 73.8165584 | | 0.01149425 | 0.69420125 |
| CSF2 | 7 | 61.875 | | 0.01075269 | 0.4464607 |
| FGF2 | 8 | 224.071807 | | 0.01162791 | 0.32409437 |
| VCAM1 | 5 | 10.0087662 | | 0.01086957 | 0.36382442 |
| CXCL2 | 7 | 6.33522727 | | 0.01 | 0.53868731 |
| CXCL10 | 8 | 13.8562951 | | 0.01041667 | 0.56230585 |
| CXCL8 | 7 | 1.32900433 | | 0.01020408 | 0.55143923 |
| SERPINE1 | 7 | 36.3041847 | | 0.01075269 | 0.54202478 |
| CXCL6 | 3 | 0 | | 0.00819672 | 0.2127898 |
| CCN2 | 7 | 39.5206349 | | 0.01 | 0.20091607 |
| CCL2 | 10 | 7.54902597 | | 0.01052632 | 0.71688373 |
| NRP1 | 3 | 144 | | 0.00854701 | 0.03973524 |
| NRP2 | 4 | 57.5 | | 0.00662252 | 0.00560052 |
| SEMA3A | 3 | 18.5 | | 0.00657895 | 0.00553017 |
| BGN | 4 | 12.3768939 | | 0.01075269 | 0.18431969 |
| ITGA10 | 2 | 1.5 | | 0.00694444 | 0.01523884 |
| ITGA6 | 2 | 39 | | 0.00884956 | 0.03909447 |
| CCN1 | 2 | 0 | | 0.00724638 | 0.03769081 |
| SPP1 | 3 | 1.33333333 | | 0.00900901 | 0.06382146 |
| ITGAV | 4 | 53.4791667 | | 0.00909091 | 0.04859927 |
| MMP7 | 3 | 7.625 | | 0.01052632 | 0.16464315 |
| LTBP1 | 5 | 15.7011905 | | 0.00943396 | 0.11706129 |
| COL1A1 | 4 | 26.5541667 | | 0.00909091 | 0.07996273 |
| IL12RB2 | 2 | 0 | | 0.00884956 | 0.14218581 |
| CNTFR | 2 | 0 | | 0.00884956 | 0.14218581 |
| IL7 | 2 | 0 | | 0.00952381 | 0.17145335 |
| CSF1 | 1 | 0 | | 0.00757576 | 0.05292033 |
| PLXNA4 | 2 | 0 | | 0.00529101 | 0.00131935 |
| LCN2 | 2 | 0 | | 0.01041667 | 0.1570782 |
| LAMA4 | 1 | 0 | | 0.00657895 | 0.00463399 |
| THBS3 | 1 | 0 | | 0.00671141 | 0.00576062 |
| IGFBP7 | 4 | 15.6541126 | | 0.00943396 | 0.22287564 |
| IL1R2 | 2 | 0 | | 0.00884956 | 0.21418897 |
| PDGFB | 1 | 0 | | 0.008 | 0.03841588 |
| COL5A2 | 3 | 8.84356061 | | 0.00917431 | 0.14985921 |
| NRCAM | 1 | 0 | | 0.00526316 | 0.00066385 |
| **Supplementary Table 3B. Network Metrics** | | | | | |
| **Metric** | | | **Value** | | |
| Clustering Coefficient | | | 0.56290484 | | |
| Number of Communities | | | 6 | | |
| Diameter | | | 6 | | |
| Radius | | | 3 | | |
| Density | | | 0.12073171 | | |

Alfaro-García et al., 2025
