# Supplementary material for: Molecular analysis and computational modeling reveal temporally separable responses triggered by DENV-induced soluble factors in endothelial cells
Source: PLoS One. 2026 Jul 31;21(7):e0354877. doi: 10.1371/journal.pone.0354877 (PMC13426972; doi:10.1371/journal.pone.0354877)
Supplement: S10 Table — (DOCX) [file pone.0354877.s025.docx]

**Supplementary Table 10. qPCR Primer sequences**

| Target | Sense | Sequence | TM (°c) | Measured MW |
| --- | --- | --- | --- | --- |
| *SNA1* | Forward | 5’-CCAGTGCCTCGACCACTATG-3’ | 62.5 | 6042.2 |
|  | Reverse | 5’-CTGCTGGAAGGTAAACTCTGG-3’ | 61.3 | 6485.3 |
| *TWIST1* | Forward | 5’-CTTCTCGGTCTGGAGGATGG-3’ | 62.5 | 6174.3 |
|  | Reverse | 5’-CCACGCCCTGTTTCTTTGAA-3’ | 58.4 | 6062.7 |
| *VIM* | Forward | 5’-GGACCAGCTAACCAACGACA-3’ | 60.5 | 6131.7 |
|  | Reverse | 5’-TCCTCCTGCAATTTCTCCCG-3’ | 59.5 | 5594.3 |
| *ACTB* | Forward | 5’-ACAGAGCCTCGCCTTTGCC-3’ | 61.7 | 5714.2 |
|  | Reverse | 5’-GAATCCTTCTGACCCATGCCC-3 | 63.3 | 6271.2 |
