# Supplementary material for: Molecular analysis and computational modeling reveal temporally separable responses triggered by DENV-induced soluble factors in endothelial cells
Source: PLoS One. 2026 Jul 31;21(7):e0354877. doi: 10.1371/journal.pone.0354877 (PMC13426972; doi:10.1371/journal.pone.0354877)
Supplement: S11 Table — (DOCX) [file pone.0354877.s026.docx]

**Supplementary Table 11. Thresholds and modulators in the advanced Boolean rules to describe the effect of CMDV on endothelial cells**

| Type | Name | Value |
| --- | --- | --- |
| Thresholds | THR_IL6 | 2 |
|  | THR_IL6 | 22 |
|  | THR_IL6 | 24 |
|  | THR_FGF2 | 24 |
|  | THR_NRP1 | 24 |
| Modulators | MOD_IL12RB2 | 1 |
|  | MOD_CNTFR | 1 |
|  | MOD_IL6 | 1 |
|  | MOD_VCAM1 | 3 |
|  | MOD_IL7 | 2 |
|  | MOD_CCL2 | 2 |
|  | MOD_CXCL10 | 2 |
|  | MOD_CXCL8 | 2 |
|  | MOD_IL1A | 2 |
|  | MOD_CXCL2 | 2 |
|  | MOD_PTGS2 | 2 |
|  | MOD_CXCL6 | 2 |
|  | MOD_CSF2 | 2 |
|  | MOD_SEMA3A | 1 |
|  | MOD_PLXNA4 | 1 |
|  | MOD_LTBP1 | 2 |
|  | MOD_THBS3 | 2 |
|  | MOD_ITGA10 | 2 |
|  | MOD_SPP1 | 2 |
|  | MOD_MMP7 | 2 |
|  | MOD_CCN2 | 2 |
|  | MOD_IGFBP7 | 2 |
|  | MOD_SERPINE1 | 24 |
|  | MOD_IL1R2 | 3 |
|  | MOD_COL5A2 | 24 |
