# Supplementary material for: Molecular analysis and computational modeling reveal temporally separable responses triggered by DENV-induced soluble factors in endothelial cells
Source: PLoS One. 2026 Jul 31;21(7):e0354877. doi: 10.1371/journal.pone.0354877 (PMC13426972; doi:10.1371/journal.pone.0354877)
Supplement: S1 File — (DOCX) [file pone.0354877.s027.docx]

**Supplemental information to**

**Molecular Analysis and Computational Modeling Reveal Temporally Separable Responses triggered by DENV-Induced Soluble Factors in Endothelial Cells**

Jenny Paola Alfaro-García^1^, Julieta M. Ramírez-Mejía^2^, Paola Rojas-Estevez^3^, Diego A. Álvarez-Díaz^4^, Geysson Javier Fernández^5^, Carlos Alberto Orozco-Castaño^2^, Boris Anghelo Rodríguez-Rey^6^, Juan Carlos Gallego-Gómez^1^* and Miguel Vicente-Manzanares^7^*

^1^ Grupo Medicina de Translación—Facultad de Medicina, Universidad de Antioquia, Medellín 050010, Colombia; jenny.alfaro@udea.edu.co

^2^ Grupo de Biología del Cáncer—Instituto Nacional de Cancerología, Bogotá 111511, Colombia; [jramirez@cancer.gov.co](mailto:jramirez@cancer.gov.co);

^3^ Grupo de Genómica de Microorganismos Emergentes—Dirección de Investigación en Salud Pública, Instituto Nacional de Salud, Bogotá 111321, Colombia; crojas@ins.gov.co

^4^ Grupo de Investigación y Desarrollo en Vacunas y Biológicos Estratégicos en Salud Pública—Dirección de Producción, Instituto Nacional de Salud, Bogotá 111321, Colombia; dalvarezd@ins.gov.co

^5^ Biología y Control de Enfermedades Infecciosas—Corporación Académica para Estudio de Patologías Tropicales, Universidad de Antioquia, Medellín 050010, Colombia; geysson.fernandez@udea.edu.co

^6^ Grupo de Fundamentos y Enseñanza de la Física y los Sistemas Dinámicos—Universidad de Antioquia, Medellín 050010, Colombia; boris.rodriguez@udea.edu.co

^7^ Molecular Mechanisms Program, Centro de Investigación del Cáncer, Instituto de Biología Molecular y Celular del Cáncer, Consejo Superior de Investigaciones Científicas (CSIC)—University of Salamanca, 37007 Salamanca, Spain

* These authors contributed equally to this work

**Correspondence:**

carlos.gallego@udea.edu.co +57 (604) 219 69 24

miguel.vicente@csic.es; miguel.vicente@usal.es +34-923294806

**Extended Results**

**2.4. Building a Non-Directed Asynchronous Model of the effect of CMDV on endothelial cell behavior (NDAM-CMDV)**

The interactome described earlier was formalized into a set of Boolean rules (Table A). To achieve this, we used a bottom-up approach to determine the expression of unknown genes at 48 hours or 120 hours (Supplementary Table 2). The data had to describe the function of the gene (node). Additionally, it considered the neighboring clusters and their interactions. Potential edge directionality was inferred from bibliographic data, which is reflected in the basic model rules. The rationale behind this choice is the limited amount of experimental data. Validation of the use of such approach as a fully predictive tool will require additional experimental validation to confer full significance to the edges. However, to ensure that the model corresponds to the experimental data, some new interactions between selected nodes were incorporated.

**Table A.** Algebra Boolean Rules for CMDV Non-directed network.

| **Node** | **Rule** |
| --- | --- |
| STAT4 | STAT4 |
| IL12RB2 | STAT4 ∧ ¬IL6 |
| CNTFR | STAT4 ∧ ¬IL6 |
| IL6 | STAT4 ∨ FN1 ∨ IL1A |
| VCAM1 | PTGS2 ∧ CXCL1 ∧ IL6 ∧ FN1 |
| IL7 | IL6 ∧ CSF2 |
| CXCL1 | IL6 ∧ ¬FGF2 |
| CCL2 | (VCAM1 ∧ IL1A ∧ CXCL2 ∧ CXCL10 ∧ CXCL1 ∧ CXCL8 ∧ IL6) ∧ ¬SERPINE1 |
| CXCL10 | CXCL1 ∧ CXCL6 ∧ CXCL2 ∧ CXCL8 ∧ IL6 |
| CXCL8 | PTGS2 ∧ IL6 ∧ CXCL1 ∧ CXCL2 |
| IL1A | (CXCL2 ∧ CXCL1 ∧ CXCL8 ∧ CXCL10 ∧ IL6) ∧ ¬SERPINE1 |
| CXCL2 | CXCL1 ∧ IL6 |
| PTGS2 | IL6 ∧ CXCL1 ∧ CCN2 |
| CXCL6 | CXCL2 ∧ CXCL1 |
| CSF2 | (IL1A ∧ IL6 ∧ CCL2 ∧ CXCL1) ∨ ¬FGF2 |
| CSF1 | CSF2 |
| FGF2 | FGF2 |
| NRP1 | FGF2 |
| SEMA3A | NRP1 ∨ NRP2 |
| NRP2 | ¬NRP1 |
| PLXNA4 | NRP2 ∧ SEMA3A |
| FN1 | ¬FGF2 |
| LCN2 | FN1 ∨ IL6 |
| BGN | FN1 ∨ IL6 |
| COL1A1 | BGN ∧ ITGA10 ∧ FN1 |
| ITGA6 | ¬FN1 |
| LAMA4 | ITGA6 |
| LTBP1 | FN1 ∨ (CCN2 ∧ CCN1) |
| ITGAV | SPP1 ∧ FN1 |
| THBS3 | ITGAV |
| ITGA10 | ITGAV |
| SPP1 | MMP7 ∧ FN1 |
| MMP7 | IL6 ∧ FN1 |
| CCN2 | FN1 ∨ SERPINA1 ∨ LTBP1 |
| CCN1 | CCN2 |
| IGFBP7 | LTBP1 ∧ IL6 ∧ CXCL10 ∧ CCN2 |
| SERPINE1 | IL6 ∧ FGF2 ∧ ¬CCL2 ∧ ¬IL1A |
| IL1R2 | IL1A ∨ IL6 |
| PDGFB | ¬FGF2 |
| COL5A2 | IL6 ∧ BGN ∧ ¬COL1A1 |
| NRCAM | ¬NRP2 |

NOT = ¬, AND=^ and OR=v

These new interactions included STAT4 with CNTFR [33, 34]; IL6 with IL1R2 [35-37], MMP7 [38], SERPINE1 [39], IGFBP7 [40, 41], COL5A2 [42-44], LCN2 [45, 46] and BGN [47]. We also included the newly described interaction between BGN and COL5A2 [48]; and of CXCL1 with VCAM1 [49] and PTGS2 [50]. Additionally, we created an edge between FN1 and MMP7 [51] and a loop among LTBP1, CCN1 and CCN2 [52]. The latter also interacts with PTGS2 [53]. Additionally, an edge was defined among LTBP1, IGFBP7 [53] and SERPINE1 [54]. SERPINE1 also interacts with IL1 [55] and FGF2 [56]. We also included the interaction of IGFBP7 with CXCL10 [57]. The resulting network consists of 41 nodes and 99 edges; however, the average clustering coefficient decreases to 0.56. The longest path (diameter) in the network between two nodes encompasses 6 edges, while the shortest path (radius), from the central node to a peripheral one, consists of 3 edges (Supplementary table 3B). Community analysis identified six highly interconnected clusters (see Table B). However, those communities are not well-defined (Mod=0.44) because they are not isolated; rather, there is a uniform interaction among them. This is due to the low connectivity between nodes (density=0.1207), which range from highly connected to poorly connected (assortativity=-0.1176).

**Table B. CMDV network communities**

| **Communities** | **Nodes** |
| --- | --- |
| Community 1 | STAT4, IL6, IL12RB2, CNTFR |
| Community2 | FN1, BGN, ITGA10, ITGA6, SPP1, ITGAV, MMP7, COL1A1, LCN2, LAMA4, THBS3 and COL5A2 |
| Community3 | IL1A, PTGS2, CXCL1, VCAM1, CXCL2, CXCL10, CXCL8, SERPINE1, CXCL6, CCL2 and IL1R2 |
| Community4 | CSF1, CSF2, FGF2, IL7 and PDGFB |
| Community5 | CCN2, CCN1, LTBP1 and IGFBP7 |
| Community6 | NRP1, NRP2, SEMA3A, PLXNA4 and NRCAM |

On the other hand, centrality analysis (Supplementary Table 3A) confirmed that IL6 is a central node because it interacts with 22 nodes, occurs 312.78 times in the shortest path between nodes (betweenness), and has a maximum eigenvector (1), even though its closeness in the network is low (0.13).

A second central node is FN1, but its interacting nodes (12) are fewer than IL6. Betweenness was 290.81 but its eigenvector was low (0.33). However, closeness was similar to that of IL6. Other relevant nodes include CXCL1, IL1α, FGF2, and CCL2. However, centralities vary. For example, FGF2 interacts with 8 nodes, and its betweenness is 224.10, but its eigenvector (0.33) is lower than that of IL1α, which interacts with 11 nodes but has a betweenness of 13.8 and an eigenvector of 0.8. This indicates that IL1α is more relevant in the present framework.

In order to enable simulations in the model, we implemented thresholds and modulators using the SPIDDOR R Studio package. These regulators can be used to modify the network generally or locally [58]. Before imposing arbitrary values, we needed to determine how many iterations would be required to reach a minimum threshold of FN1 degradation. To do this, we used a first-order modified model with regular catabolic degradation rate [59].

FN1 required 23 iterations to reach the minimum threshold. The maximum thresholds and modulators are 24, establishing 23 FN1-dependent interactions. This was regularly used to induce a state change in a particular node. The only exception was IL1R2, which displayed a threshold of 22, allowing the node to maintain its presence in the model.

Other regulators influence the network in initial stages and are defined by how much time a cell takes to perform transcription, translation, or metabolic synthesis. The first two processes can be assumed to be relatively quick and comparable to each other [60]. Also, CMDV contains some proteins corresponding to specific nodes, for example IL6 [15], which increases the cellular response rate. However, metabolic synthesis involves multiple steps of diverse duration. An example is VCAM1, which is produced by endothelial cells in response to inflammation [61, 62]. This introduces an asynchronous behavior component in the system, which better simulates the experimental results.

**2.5. 2.5. NDAM-CMDV robustness, accuracy and attractors**

The accuracy and robustness of the model in describing the experimental data were then evaluated, taking the above-described conditions and parameters into consideration. 25000 simulations, each consisting of 100 iterations with asynchronous activation, were performed **(Figure 6A and Supplementary Table 4)**. The system exhibited the expected behavior: the pro-inflammatory and endothelial dysfunction nodes were activated during the first 25 iterations and then turned off. Some nodes (CSF1, CSF2, ITGA6, LAMA4, and NRCAM) displayed a mildly nonspecific (noisy) signal during the first three iterations. However, these did not exceed an activation state of 0.5, thereby did not impair the proper evolution of the system. The model is built to correct such disturbances by stabilizing the state of these nodes ([dx]((t) )/dt=0) until they become activated. In this manner, such behavior preserves the dynamics of the network.

The activation percentage, media, and standard deviation from each node is highly predictable because they exhibit high activation but low variance (STAT4, FGF2, IL6, NRP1, SEMA3A, BGN, and LCN2). This means these nodes are consistent across the entire system. Another subgroup of nodes displays low means and variance (IL1A, CCL2, CSF2, CSF1, and IL7). Finally, another high predictability group includes those with high means and medium variance (CCN1, CCN2, LTBP1, and IL1R2), indicating that they are activated and tend to be consistent.

However, other nodes tend to be more unpredictable due to their high variance and average means (SERPINE1, COL5A2, NRCAM, LAMA4, and ITGA6), or high variance and low means (IL12RB2, NRP2, CNTFR, PDGFB, FN1, and PLXNA4).

On the other hand, we also determined the system attractors (Supplementary Table 5). The analysis identified them using various approaches, including synchronic and asynchronous methods, thereby identifying synchronic and asynchronous attractors. In the first two analyses, attractors were consistent (STAT4, IL6, FGF2, NRP1, SEMA3A, LCN2, BGN, ITGA6, LAMA4, LTBP1, CCN1/2, SERPINE1, IL1R2, COL5A2, and NRCAM), suggesting that these nodes have influenced the dynamic behavior of the system. However, this analysis identified 31 nodes with a high recurrence. This strongly indicates that the model may be misaligned. Nevertheless, eight attractors (IL6, SEMA3A, LCN2, BGN, LTBP1, CCN1, CCN2, and IL1R2) were common in all the analyses. These indicate that the network has a moderate degree of complexity, high stability, and predictability, which are strong indicators of robustness. Additionally, the accuracy of the CMDV model against the experimental data is good, indicating its potential to study endothelial dysfunction in dengue using a cellular approach.
