# Supplementary material for: Molecular analysis and computational modeling reveal temporally separable responses triggered by DENV-induced soluble factors in endothelial cells
Source: PLoS One. 2026 Jul 31;21(7):e0354877. doi: 10.1371/journal.pone.0354877 (PMC13426972; doi:10.1371/journal.pone.0354877)
Supplement: Attachment — Submitted filename: point-by-point.pdf [file pone.0354877.s029.pdf]

***POINT BY POINT REPLY TO THE REVIEWERS***

*Reviewer #1*

**1. Why are the immunofluorescent images shown in black and white, and only the merged images and showed in colors? In some images, it is difficult to appreciate details of the fluorescent mark. Please, show all the panels in full color.**

Fluorescent images are computer-colored because the sensors of most CCD or CMOS only detect intensity, not color. However, we agree that white on black images are not as clear as they could be. Hence, we have replaced the white on black color panels with black on white panels, which are much easier for the human eye to observe. We have elected to preserve the co-localization, red-blue in panels (A) and (B) of Figures 1-2 and S1-S2; and red-blue-green in panels (C) of the same figures.

**2. Image 1C: It is not possible to differentiate the SNAIL mark; this should be a green mark according to the described methodology.**

While the changes implemented in response to question #3 have improved visualization of SNAIL, its staining was not particularly intense at 48h. This is the main reason the colocalization images do not clearly show green signal in panel C. In fact, SNAIL staining in HMEC-1 cells does not appear to be a widely used marker of EndMT in the literature. However, it is in other cell types (REF). Consistently, the signal is much clearer at 120h, particularly in nuclear dots (Figure 2C, TGFbeta panel). Interestingly, the distribution of SNAIL in CMDV-treated cells at 120h was different from that induced by TGFbeta, with a mainly perinuclear distribution. We have included this in the discussion. In any case, we confirmed the increased SNAIL levels by semi-quantitative PCR (Figure 4) and in-cell Western blot (Alfaro et al., 2025, doi: 10.3390/ijms26052139)

**3. Is it possible to include some description of the cytopathic effect, if any, observed between controls and CMDV-exposed cells?**

We now refer to the data requested by the reviewer, which appeared in Alfaro et al., 2025 as a Supplementary Figure 1 (doi: 10.3390/ijms26052139). Perhaps the most crucial argument is that concentrated CMDVs do not produce PFCs, illustrating the lack of active viral infection under these conditions. The point we try to make is that the effects observed are due to the soluble factors contained in the media, which may be of viral, or cellular, origin.

**4. It is not clear how the authors determined the imatinib concentration used in their experiments. Please include this information.**

The dose of imatinib used was determined experimentally in a peer-reviewed publication that we now cite in the Material and Methods section to this effect (doi: 10.3390/v15071437).

**5. Line 501: Please clarify why the cell culture media was changed every 24 hours? and specify if every 24 h the treatments were renewed or not.**

The main reason is because TGFbeta degrades over time (doi: 10.1007/978-1-4939-2966-5_25). Refreshing the media with fresh TGFbeta allowed us to obtain a robust mesenchymal phenotype. For consistency, cells exposed to CMDV were also restimulated every 24h.

**6. Line 479: Please specify 1% of what.**

It was the percentage of FBS %. Corrected as required.

**7. Suggestion: incorporate to the discussion if the plasma leakage or hemorrhage has been observed in patients infected with all dengue virus serotypes or are more associated with a specific serotype or more predominantly in serotype 2 used in this work, or any other information that can help to enrich the context of this particular topic**

"The four serotypes of dengue produce the same disease; the difference is in the symptoms apparition or intensity; all can evolve to SD but DENV2 has been extensively associated with this complication and a higher mortality rate". (doi: 10.1017/s0950268820000229 ; doi: 10.1371/journal.pntd.0006817 ; doi: 10.1038/s41598-024-67553-1 ; doi: 10.7759/cureus.15029; 10.1186/s40001-023-01454-3 ).

**8. -Lanes 435-438: The authors: "...we demonstrate that dengue infection can trigger a reversible EndMT-like program in endothelial cells, linking the acute inflammatory surge to transient vascular leakage". Comment: according to the data and experiments presented in this work, it is too strong to state "we demonstrated that dengue infection...", due experimentally only one cell line and only one serotype of dengue virus were**

**used. I suggest scaling down this conclusion and focusing it on the experiments carried out.**

We have toned down this conclusion as indicated below:

“In conclusion, we demonstrate that **soluble factors** from dengue infection can **promote** a reversible EndMT-like **mechanism** in endothelial cells, linking the acute inflammatory surge to transient vascular leakage”

**Reviewer #2**

**1. The EndMT claim is overstated. The data support partial, reversible endothelial–mesenchymal changes, but not full EndMT. The manuscript should clearly define "partial EndMT/EndMT-like," use this term consistently, and explicitly acknowledge that key functional EndMT assays (migration, angiogenesis, in vivo validation) are not included.**

We agree and have reframed all the mentions to partial EndMT/EndMT-like throughout the text.

**2. The link between RNAseq results and the Boolean network is not sufficiently clear. The authors should specify how DEGs were selected as nodes, which nodes/edges are directly supported by their data versus literature only, and provide a concise biological justification for the choice of a non-directed asynchronous model.**

Thank you for this observation. In response to this comment, we have expanded the description of the network construction and the modeling paradigm to clarify the integration of our transcriptomic data with established literature. This has been incorporated into the Material and Methods section, under heading 4.9.

Node Selection and Edge Support

The nodes included in our model were prioritized from the differentially expressed genes (DEGs) identified at 48 and 120 hours post-exposure to CMDV. The selection and initial interaction mapping were performed using Cytoscape and STRING-db, with the latter providing the primary experimental and bibliographic support for each initial edge. While most nodes originate directly from our DEGs, some nodes presented "unknown" or inconsistent states at specific time points (e.g., at 48h vs. 120h). In these cases, we performed an extensive bibliographic search to determine the most probable biological state, ensuring the model remained consistent with the observed cellular response. Additionally, we manually verified interactions not initially detected by STRING-db or Cytoscape through literature review. These suggested interactions, which refine the molecular mechanisms affected by CMDV, are explicitly highlighted as predictions that require future experimental validation to increase the model's robustness.

Biological Justification for the Asynchronous Model

We selected a non-directed asynchronous paradigm because it more accurately reflects real biological scenarios compared to synchronous models. In a biological system, it is extremely rare for all molecular events to occur simultaneously.

Asynchronous modeling assumes that variables are updated one at a time, capturing the stochastic nature of cellular processes. This is particularly relevant for describing multi-stability—the simultaneous presence of multiple state-space attractors (e.g., epithelial vs. mesenchymal states)—which describes how a single viral stimulus can lead to different cellular fates. Then, biological functions operate on vastly different timescales (e.g., fast protein-protein interactions vs. slower protein synthesis). Asynchronous and stochastic updating help mitigate the temporal distortions of synchronous models, allowing the representation of hierarchical dependencies, such as the requirement for a protein to be synthesized before it can be phosphorylated. In this models "complex attractors" emerge as overlapping loops in the state graph. This complexity is better suited for capturing system-wide biological behavior and cellular plasticity, as the trajectory of these graphs defines stable states (attractors) that represent biological equilibrium under specific conditions (10.3390/cells10112863; 10.1016/j.csbj.2020.03.001).

**3. IL-6 and FN1 are presented as central mechanistic nodes largely based on modeling. Direct perturbation experiments (e.g., IL-6 neutralization, FN1 knockdown/inhibition under CMDV exposure) are needed to test predictions on inflammatory genes, mesenchymal markers, and junctional integrity; if not feasible, claims should be softened and the model explicitly framed as hypothesis-generating.**

We appreciate the feedback and acknowledge that this is a limitation of our work, which is why we emphasize the urgent need for experimental validation. Therefore, we have redefined the model to a hypothesis generator. We have reflected this in lines 436-442.

**4. The temporal interpretation relative to severe dengue needs sharpening. The authors should more explicitly map the 48 h (inflammatory) and 120 h (repair/ECM) phases to clinical stages (peak viremia vs defervescence/plasma leakage) and emphasize a small set of early vs late genes with known roles in vascular leakage.**

This is a very good suggestion that we have implemented in page 5, lines 140-143 of the Results section. At 48h, we observe a pro-immune cell migration profile, with induction of genes such as VCAM-1 (promotes neutrophil, monocyte and lymphocyte/NK cell extravasation), CXCL1 (chemoattractant for neutrophils), CCL2/CCL6 (chemoattractant for monocytes) and multiple growth factors (FGF, PDGF, IL6) that favor endothelial permeability. On the other hand, genes induced at 120h include MYC, which could mark the beginning of the endothelial repair phase together with the observed induction of TENM2 (adhesive scaffold), and specific

types of collagen (COL5A2). A key issue here, which we now include in the discussion, is that overdoing TENM2 signaling may trigger hypercontraction via RhoA/ROCK, increasing endothelial permeability instead of decreasing it. Thank you for this very constructive and interesting question.

**5. CMDV preparation and controls require clearer description. Residual infectivity after UV inactivation should be explicitly ruled out, the generation and treatment of control conditioned media clarified, and the relationship between CMDV cytokine/NS1 levels and reported patient plasma levels discussed where possible.**

Thank you for these constructive observations. We have addressed each point by adding details and references to the Material and Methods section (page 18, section 4.2, lines 471-482). The discussion of the relationship between CMDV/NS1 levels and reported patient plasma levels has been added to the Discussion (page 15, lines 394-410).

**6. Parts of the Results (especially RNAseq and modeling sections) are overly detailed and repetitive. The main text should focus on biologically important patterns, with most graph-theoretic detail, full Boolean rule sets, and some DEG/GO listings moved to Supplementary material.**

Following this suggestion, we have lightened up sections 2.4 and 2.5 and reorganized figures into Supplemental as indicated. This is now included into a new Supplementary information file.

**7. The manuscript would benefit from a clearer articulation of novelty relative to earlier CMDV/imatinib work (refs 13–16), emphasizing the added value of global transcriptomics, biphasic response characterization, and formal network modeling.**

Thanks for this suggestion. We have included this in page 4, lines 92-98. Also, we have highlighted this by modifying the title of the manuscript.

**8. Statistical reporting needs standardization: clearly state per figure which tests were used, how assumptions were checked, and how multiple comparisons were handled; harmonize or justify the use of FDR vs nominal p-value thresholds in DEG and network construction.**

Response: We agree and have revised the Methods accordingly. For RNA-seq, we added a short QC paragraph describing trimming and alignment QC, sample-level exploratory analysis (variance-stabilized PCA / clustering), and the criteria used to exclude low-quality samples (none were excluded under the stated thresholds). For DEGs, we now state the exact thresholds (FDR and effect size) and confirm the directionality of contrasts. For immunofluorescence, we expanded the quantification description to specify the outcome metrics quantified in ImageJ, how many fields/images and cells were analyzed per replicate, and how observer bias was minimized (randomized file names/blinded scoring during quantification). Finally, we condensed the Statistics subsection to avoid redundancy and relocated test-specific details to figure legends.

<u>Inmunofluorescence:</u>

*Revised text (Methods – immunofluorescence quantification; Section 4.4; lines KK–LL): "To ensure objective quantification and minimize observer bias, all image files were randomized and blinded before analysis. Data processing was performed using ImageJ version 1.54f, where the Integrated Density (IntDen) for both red and green channels was recorded. To maintain data quality, an outlier removal step was implemented using the Interquartile Range (IQR) method (values outside Q1-1.5\times IQR and Q3+1.5\times IQR were excluded). Given that the data did not meet the assumptions of normality (Shapiro-Wilk test) or homogeneity of variance (Bartlett's test), a non-parametric Kruskal-Wallis test followed by a Dunn's post-hoc test with Bonferroni correction was used for statistical comparisons. Final fluorescence intensity values were expressed as a percentage of expression relative to the total intensity to account for variations in cell density across treatments. Analysis was conducted using R software (version 4.3.0) with at least three biological replicates per condition."*

*Revised text (Methods – RNA-seq QC; Section 4.5; lines II–JJ): "After trimming (Trimmomatic) and read-level QC (FastQC), we assessed sample-level QC using variance-stabilized counts (DESeq2) and performed principal component analysis and hierarchical clustering to confirm replicate concordance and to identify potential outliers. No samples met exclusion criteria based on low library complexity or abnormal mapping/QC metrics."*

**9. Figures could better support key claims: include a simplified network diagram highlighting IL-6 and FN1; a clear visualization of the biphasic**

**inflammatory vs repair response (CMDV vs TGF-β1, 48 h vs 120 h); and a simple centrality plot for top hub nodes.**

We have enhanced the visualization of specific figures to comply with the reviewer's request: briefly, we have modified the network by color-coding the genes as inflammatory only (induced by CMDV at 48h), reparative only (induced by CMDV at 120h) and always on (induced at both 48h and 120h). We have also modified the size of the nodes and made them proportional to their centrality. As for the biphasic response, we have included a simplified version of the figure with a representative example of the profiles obtained in the simulations, their meaning and the genes that show the same behavior. Hope this makes the figures provide better visual support for the key claims of this part of the study.

**10. Results subsections should be streamlined by summarizing DEG counts and GO terms in a compact table or schematic and avoiding repeated listing of similar gene sets.**

We strongly believe that it is important that the readership of the article have full access to the entire dataset at the tip of their fingers. While data is accessible as described in the Material and Methods section, we believe that the graphs included in the Supplemental Figure/Table section constitute a more precise and scientifically sound approach compared to summary tables and schematics. However, we have streamlined the description of these data in the Results section.

**11. Methods would be clearer with brief notes on RNAseq quality control (e.g., PCA, clustering), exact criteria for calling DEGs, details of immunofluorescence quantification (metrics, blinding), and a more concise, focused statistical methods section.**

We agree and have standardized statistical reporting across the manuscript. We revised the Statistical Analysis subsection and updated each figure legend to explicitly state: (i) the statistical test used; (ii) the unit of replication (biological vs technical); (iii) the normality/variance checks performed (or the reason for choosing non-parametric tests); and (iv) the multiple-comparison correction method where applicable. We also clarified that DEG calling is performed with DESeq2 using Benjamini–Hochberg FDR control (FDR < 0.05), and we now use a consistent adjusted-threshold framework when selecting gene sets for downstream analyses. Where nominal p-values are displayed (e.g., enrichment outputs), we now report adjusted values. Network construction is now described as based on FDR-filtered DEGs, with literature-supported edges documented separately (Suppl. Table 2) rather than being conflated with data-derived support.

*Revised text (Methods – DEGs and multiple testing; Section 4.5; lines 529–532): "Differential expression was performed with DESeq2; p-values were adjusted using the Benjamini–Hochberg procedure and genes were considered significant at FDR < 0.05 with |log2FC| ≥ 1. All downstream RNA-seq-derived gene sets used for modeling were restricted to this FDR-controlled list."*

*Revised text (Methods – statistics and figure legends; lines GG–HH): "For microscopy quantifications and qPCR validations, we report the statistical test in each figure legend, after assessing distributional assumptions (Shapiro–Wilk for normality; Levene's test for homoscedasticity). When assumptions were not met, we used the corresponding non-parametric alternative (Mann–Whitney or Kruskal–Wallis with Dunn's post hoc). Multiple comparisons were controlled using Benjamini–Hochberg where relevant."*

**12. Language and style require moderate editing: correct grammatical errors ("EndMT is defines as..."), shorten long sentences, reduce repetitive wording, and standardize terminology (e.g., "EndMT-like" vs "EndMT") to improve readability.**

We appreciate the comment. We have gone through the entire text and corrected as many typos, grammatical errors and repetitions as possible.
