# Supplementary material for: Molecular analysis and computational modeling reveal temporally separable responses triggered by DENV-induced soluble factors in endothelial cells
Source: PLoS One. 2026 Jul 31;21(7):e0354877. doi: 10.1371/journal.pone.0354877 (PMC13426972; doi:10.1371/journal.pone.0354877)
Supplement: Attachment — Submitted filename: rebuttal to the reviewer R2.docx [file pone.0354877.s030.docx]

**POINT-BY-POINT RESPONSE TO THE EDITOR AND REVIEWER’S COMMENTS**

1. Reviewer query: To facilitate reader analysis and interpretation, we recommend presenting the results as clearly as possible. Specifically, for the Immunofluorescence data, we suggest displaying all panels in color, (nor only the merge panels) as this will significantly improve the readability and assessment of the expression patterns. Standardize all the immunofluorescence images of the article in colors.

Rephrased by the Editor: The reviewer considers that the manuscript needs to standardize all immunofluorescence images to color

**Response: We have replaced the black and white images in Figures 1, 2, S1 and S2, with color images as requested.**

2. The authors use the term SNAIL and SNA1 (Fig. 4) as the same? Please clarify and standardize.

**Response: As requested, we have standardized the use of Snail to designate protein and *SNA1* (cursive) to designate the coding gene. This has been extended to other genes in the manuscript, e.g. vimentin (protein) vs. *VIM* (gene) and N-cadherin (protein) vs. *CDH2* (gene).**
