# Supplementary material for: Molecular analysis and computational modeling reveal temporally separable responses triggered by DENV-induced soluble factors in endothelial cells
Source: PLoS One. 2026 Jul 31;21(7):e0354877. doi: 10.1371/journal.pone.0354877 (PMC13426972; doi:10.1371/journal.pone.0354877)
Supplement: Peer review history

# Peer review: Decision Letter 0

**DOI:** 10.1371/journal.pone.0354877.r001

**Authors:** Victoria Pando-Robles

2 Feb 2026

PONE-D-25-67733

Molecular and Computational Insights into the Mechanism of Endothelial Dysfunction Induced by Soluble Factors during Dengue Virus Infection

PLOS One

Dear Dr. Vicente-Manzanares,

Thank you for submitting your manuscript to PLOS ONE. After careful consideration, we feel that it has merit but does not fully meet PLOS ONE’s publication criteria as it currently stands. Therefore, we invite you to submit a revised version of the manuscript that addresses the points raised during the review process.

Please submit your revised manuscript by Mar 19 2026 11:59PM. If you will need more time than this to complete your revisions, please reply to this message or contact the journal office at plosone@plos.org. When you're ready to submit your revision, log on to https://www.editorialmanager.com/pone/ and select the 'Submissions Needing Revision' folder to locate your manuscript file.

Please include the following items when submitting your revised manuscript:

- A letter that responds to each point raised by the academic editor and reviewer(s). You should upload this letter as a separate file labeled 'Response to Reviewers'.
- A marked-up copy of your manuscript that highlights changes made to the original version. You should upload this as a separate file labeled 'Revised Manuscript with Track Changes'.
- An unmarked version of your revised paper without tracked changes. You should upload this as a separate file labeled 'Manuscript'.

If you would like to make changes to your financial disclosure, please include your updated statement in your cover letter. Guidelines for resubmitting your figure files are available below the reviewer comments at the end of this letter.

If applicable, we recommend that you deposit your laboratory protocols in protocols.io to enhance the reproducibility of your results. Protocols.io assigns your protocol its own identifier (DOI) so that it can be cited independently in the future. For instructions see: https://journals.plos.org/plosone/s/submission-guidelines#loc-laboratory-protocols. Additionally, PLOS ONE offers an option for publishing peer-reviewed Lab Protocol articles, which describe protocols hosted on protocols.io. Read more information on sharing protocols at https://plos.org/protocols?utm_medium=editorial-email&utm_source=authorletters&utm_campaign=protocols.

We look forward to receiving your revised manuscript.

Kind regards,

Victoria Pando-Robles, Ph.D.

Academic Editor

PLOS One

Journal Requirements:

When submitting your revision, we need you to address these additional requirements.

1. Please ensure that your manuscript meets PLOS ONE's style requirements, including those for file naming. The PLOS ONE style templates can be found at

https://journals.plos.org/plosone/s/file?id=wjVg/PLOSOne_formatting_sample_main_body.pdf and

https://journals.plos.org/plosone/s/file?id=ba62/PLOSOne_formatting_sample_title_authors_affiliations.pdf

2. We note that the grant information you provided in the ‘Funding Information’ and ‘Financial Disclosure’ sections do not match.

When you resubmit, please ensure that you provide the correct grant numbers for the awards you received for your study in the ‘Funding Information’ section.

3. Thank you for stating the following financial disclosure:

“This work was supported by Sci. Min. (Colombia) grant CTO 917-2019, Universidad de Antioquia grant CODI-2020-34137 (JCG-G) and PID2020-116232RB-I00, PID2023-153018NB-I00 and ECRIN-M3 from AECC/AIRC/CRUK to MV-M.”

Please state what role the funders took in the study.  If the funders had no role, please state: "The funders had no role in study design, data collection and analysis, decision to publish, or preparation of the manuscript." If this statement is not correct you must amend it as needed.

Please include this amended Role of Funder statement in your cover letter; we will change the online submission form on your behalf.

4. Please note that funding information should not appear in the Acknowledgments section or other areas of your manuscript. We will only publish funding information present in the Funding Statement section of the online submission form. Please remove any funding-related text from the manuscript.

5. We note that your Data Availability Statement is currently as follows:

“All relevant data are within the manuscript and its Supporting Information files.”

Please confirm at this time whether or not your submission contains all raw data required to replicate the results of your study. Authors must share the “minimal data set” for their submission. PLOS defines the minimal data set to consist of the data required to replicate all study findings reported in the article, as well as related metadata and methods (https://journals.plos.org/plosone/s/data-availability#loc-minimal-data-set-definition).

For example, authors should submit the following data:

- The values behind the means, standard deviations and other measures reported;

- The values used to build graphs;

- The points extracted from images for analysis.

Authors do not need to submit their entire data set if only a portion of the data was used in the reported study.

If your submission does not contain these data, please either upload them as Supporting Information files or deposit them to a stable, public repository and provide us with the relevant URLs, DOIs, or accession numbers. For a list of recommended repositories, please see https://journals.plos.org/plosone/s/recommended-repositories.

If there are ethical or legal restrictions on sharing a de-identified data set, please explain them in detail (e.g., data contain potentially sensitive information, data are owned by a third-party organization, etc.) and who has imposed them (e.g., an ethics committee). Please also provide contact information for a data access committee, ethics committee, or other institutional body to which data requests may be sent. If data are owned by a third party, please indicate how others may request data access.

6. When completing the data availability statement of the submission form, you indicated that you will make your data available on acceptance. We strongly recommend all authors decide on a data sharing plan before acceptance, as the process can be lengthy and hold up publication timelines. Please note that, though access restrictions are acceptable now, your entire data will need to be made freely accessible if your manuscript is accepted for publication. This policy applies to all data except where public deposition would breach compliance with the protocol approved by your research ethics board. If you are unable to adhere to our open data policy, please kindly revise your statement to explain your reasoning and we will seek the editor's input on an exemption. Please be assured that, once you have provided your new statement, the assessment of your exemption will not hold up the peer review process.

7. We notice that your supplementary figures/tables are included in the manuscript file. Please remove them and upload them with the file type 'Supporting Information'. Please ensure that each Supporting Information file has a legend listed in the manuscript after the references list.

8. If the reviewer comments include a recommendation to cite specific previously published works, please review and evaluate these publications to determine whether they are relevant and should be cited. There is no requirement to cite these works unless the editor has indicated otherwise.

**Additional Editor Comments:**

The manuscript integrates experimental and computational approaches and the topic is interesting; however, the author needs to improve the figures, clarify the EndMT claim, and reorganize results and methods.

[Note: HTML markup is below. Please do not edit.]

Reviewers' comments:

Reviewer's Responses to Questions

**Comments to the Author**

1. Is the manuscript technically sound, and do the data support the conclusions?

The manuscript must describe a technically sound piece of scientific research with data that supports the conclusions. Experiments must have been conducted rigorously, with appropriate controls, replication, and sample sizes. The conclusions must be drawn appropriately based on the data presented.

Reviewer #1: Partly

Reviewer #2: Yes

**********

2. Has the statistical analysis been performed appropriately and rigorously?

Reviewer #1: Yes

Reviewer #2: Yes

**********

3. Have the authors made all data underlying the findings in their manuscript fully available?

The PLOS Data policy requires authors to make all data underlying the findings described in their manuscript fully available without restriction, with rare exception (please refer to the Data Availability Statement in the manuscript PDF file). The data should be provided as part of the manuscript or its supporting information, or deposited to a public repository. For example, in addition to summary statistics, the data points behind means, medians and variance measures should be available. If there are restrictions on publicly sharing data—e.g. participant privacy or use of data from a third party—those must be specified.

Reviewer #1: Yes

Reviewer #2: Yes

**********

4. Is the manuscript presented in an intelligible fashion and written in standard English?

PLOS ONE does not copyedit accepted manuscripts, so the language in submitted articles must be clear, correct, and unambiguous. Any typographical or grammatical errors should be corrected at revision, so please note any specific errors here.

Reviewer #1: Yes

Reviewer #2: Yes

**********

5. Review Comments to the Author

Please use the space provided to explain your answers to the questions above. You may also include additional comments for the author, including concerns about dual publication, research ethics, or publication ethics. (Please upload your review as an attachment if it exceeds 20,000 characters)

Reviewer #1: Alfaro-García et al, present the manuscript entitled: “Molecular and Computational Insights into the Mechanism of Endothelial Dysfunction Induced by Soluble Factors during Dengue Virus Infection”. In this work, the authors investigated the key features of the exposure to endotelial cells with CMDV. The authors carry out in vitro experiments in endotelial cells and computational modeling to investigate the potential impact on endotelial dysfunction.

Comments:

-Why the immunofluorescent images are showed in black and white, and only the merged images and showed in colors?. In some images is diffcult to appreciate details of the fluorescent mark. Please, show all the panels in full color.

-Image 1C: It is not posible to differenciate the SNAIL mark, this should be a green mark according the described methodology used.

-Is it posible to include some description about the cytopathic effect, if any, observed between controls and CMDV exposed cells?

-It is not clear how the authors determined the imatinib concentration used in their experiments. Please include this information.

-Line 501: Please clarify why the cell culture media was changed every 24 hours? and especify if every 24 h the treatments were renewed or not.

- Suggestion: incorporate to the discussion if the plasma likeage or hemorrhage, has been observed in patients infected with all dengue virus serotypes or are more associated with an specific serotype or more predominantly in serotype 2 used in this work, or any other information that can help to enrich the context of this particular topic.

-Lanes 435-438: The authors: “…we demonstrate that dengue infection can trigger a reversible EndMT-like program in endothelial cells, linking the acute inflammatory surge to transient vascular leakage”.

Comment: according the data and experiments presented in this work, it is too strong to state “we demostrated that dengue infection…”, due experimentally only one cell line and only one serotype of dengue virus were used. I suggest scale down this conclusión and focus it according the experiments carried out.

Minors:

-Lines 137-141: Improve redaction

-Line 479: Please specify 1% of what.

Reviewer #2: This manuscript investigates how soluble factors released during dengue virus (DENV) infection (CMDV) drive endothelial dysfunction, combining in vitro HMEC-1 assays, bulk RNAseq, and a non-directed asynchronous Boolean network model to identify IL-6 and FN1 as central nodes in a transient EndMT-like program. The topic is timely and relevant, and integrating experimental and computational approaches is a strength, but the current version needs clarification of the EndMT claim, tighter linkage between in silico predictions and wet-lab validation, and substantial streamlining and reorganization of some Results/Methods sections before it is suitable for publication.

Major concerns

The EndMT claim is overstated. The data support partial, reversible endothelial–mesenchymal changes, but not full EndMT. The manuscript should clearly define “partial EndMT/EndMT-like,” use this term consistently, and explicitly acknowledge that key functional EndMT assays (migration, angiogenesis, in vivo validation) are not included.

The link between RNAseq results and the Boolean network is not sufficiently clear. The authors should specify how DEGs were selected as nodes, which nodes/edges are directly supported by their data versus literature only, and provide a concise biological justification for the choice of a non-directed asynchronous model.

IL-6 and FN1 are presented as central mechanistic nodes largely based on modeling. Direct perturbation experiments (e.g., IL-6 neutralization, FN1 knockdown/inhibition under CMDV exposure) are needed to test predictions on inflammatory genes, mesenchymal markers, and junctional integrity; if not feasible, claims should be softened and the model explicitly framed as hypothesis-generating.

The temporal interpretation relative to severe dengue needs sharpening. The authors should more explicitly map the 48 h (inflammatory) and 120 h (repair/ECM) phases to clinical stages (peak viremia vs defervescence/plasma leakage) and emphasize a small set of early vs late genes with known roles in vascular leakage.

CMDV preparation and controls require clearer description. Residual infectivity after UV inactivation should be explicitly ruled out, the generation and treatment of control conditioned media clarified, and the relationship between CMDV cytokine/NS1 levels and reported patient plasma levels discussed where possible.

Parts of the Results (especially RNAseq and modeling sections) are overly detailed and repetitive. The main text should focus on biologically important patterns, with most graph-theoretic detail, full Boolean rule sets, and some DEG/GO listings moved to Supplementary material.

The manuscript would benefit from a clearer articulation of novelty relative to earlier CMDV/imatinib work (refs 13–16), emphasizing the added value of global transcriptomics, biphasic response characterization, and formal network modeling.

Minor concerns

Statistical reporting needs standardization: clearly state per figure which tests were used, how assumptions were checked, and how multiple comparisons were handled; harmonize or justify the use of FDR vs nominal p-value thresholds in DEG and network construction.

Figures could better support key claims: include a simplified network diagram highlighting IL-6 and FN1; a clear visualization of the biphasic inflammatory vs repair response (CMDV vs TGF-β1, 48 h vs 120 h); and a simple centrality plot for top hub nodes.

Results subsections should be streamlined by summarizing DEG counts and GO terms in a compact table or schematic and avoiding repeated listing of similar gene sets.

Methods would be clearer with brief notes on RNAseq quality control (e.g., PCA, clustering), exact criteria for calling DEGs, details of immunofluorescence quantification (metrics, blinding), and a more concise, focused statistical methods section.

Language and style require moderate editing: correct grammatical errors (“EndMT is defines as…”), shorten long sentences, reduce repetitive wording, and standardize terminology (e.g., “EndMT-like” vs “EndMT”) to improve readability.

**********

6. PLOS authors have the option to publish the peer review history of their article (what does this mean?). If published, this will include your full peer review and any attached files.

If you choose “no”, your identity will remain anonymous but your review may still be made public.

**Do you want your identity to be public for this peer review?**  For information about this choice, including consent withdrawal, please see our Privacy Policy.

Reviewer #1: No

Reviewer #2: No

**********

[NOTE: If reviewer comments were submitted as an attachment file, they will be attached to this email and accessible via the submission site. Please log into your account, locate the manuscript record, and check for the action link "View Attachments". If this link does not appear, there are no attachment files.]

To ensure your figures meet our technical requirements, please review our figure guidelines: https://journals.plos.org/plosone/s/figures

You may also use PLOS’s free figure tool, NAAS, to help you prepare publication quality figures: https://journals.plos.org/plosone/s/figures#loc-tools-for-figure-preparation.

NAAS will assess whether your figures meet our technical requirements by comparing each figure against our figure specifications.

# Author comment: Author response to Decision Letter 1

**DOI:** 10.1371/journal.pone.0354877.r002

31 Mar 2026

We have modified the funding info to the best of our knowledge. All the scientific responses and rebuttals are including in the accompanying documents.

Attachment Submitted filename: point-by-point.pdf

# Peer review: Decision Letter 1

**DOI:** 10.1371/journal.pone.0354877.r003

**Authors:** Victoria Pando-Robles

8 Jul 2026

PONE-D-25-67733R1

Molecular Analysis and Computational Modeling Reveal Temporally Separable Responses triggered by DENV-Induced Soluble Factors in Endothelial Cells

PLOS One

Dear Dr. Vicente-Manzanares,

Thank you for submitting your manuscript to PLOS ONE. After careful consideration, we feel that it has merit but does not fully meet PLOS ONE’s publication criteria as it currently stands. Therefore, we invite you to submit a revised version of the manuscript that addresses the points raised during the review process.

=============================

The reviewer considers that the manuscript needs to standardize all immunofluorescence images to color.

=============================

Please submit your revised manuscript by Aug 22 2026 11:59PM. If you will need more time than this to complete your revisions, please reply to this message or contact the journal office at plosone@plos.org. When you're ready to submit your revision, log on to https://www.editorialmanager.com/pone/ and select the 'Submissions Needing Revision' folder to locate your manuscript file.

Please include the following items when submitting your revised manuscript:

- A letter that responds to each point raised by the academic editor and reviewer(s). You should upload this letter as a separate file labeled 'Response to Reviewers'.
- A marked-up copy of your manuscript that highlights changes made to the original version. You should upload this as a separate file labeled 'Revised Manuscript with Track Changes'.
- An unmarked version of your revised paper without tracked changes. You should upload this as a separate file labeled 'Manuscript'.

If you would like to make changes to your financial disclosure, please include your updated statement in your cover letter. Guidelines for resubmitting your figure files are available below the reviewer comments at the end of this letter.

If applicable, we recommend that you deposit your laboratory protocols in protocols.io to enhance the reproducibility of your results. Protocols.io assigns your protocol its own identifier (DOI) so that it can be cited independently in the future. For instructions see: https://journals.plos.org/plosone/s/submission-guidelines#loc-laboratory-protocols. Additionally, PLOS ONE offers an option for publishing peer-reviewed Lab Protocol articles, which describe protocols hosted on protocols.io. Read more information on sharing protocols at https://plos.org/protocols?utm_medium=editorial-email&utm_source=authorletters&utm_campaign=protocols.

As the corresponding author, your ORCID iD is verified in the submission system and will appear in the published article. PLOS supports the use of ORCID, and we encourage all coauthors to register for an ORCID iD and use it as well. Please encourage your coauthors to verify their ORCID iD within the submission system before final acceptance, as unverified ORCID iDs will not appear in the published article. *Only*  the individual author can complete the verification step; PLOS staff *cannot*  verify ORCID iDs on behalf of authors.

We look forward to receiving your revised manuscript.

Kind regards,

Victoria Pando-Robles, Ph.D.

Academic Editor

PLOS One

Journal Requirements:

If the reviewer comments include a recommendation to cite specific previously published works, please review and evaluate these publications to determine whether they are relevant and should be cited. There is no requirement to cite these works unless the editor has indicated otherwise.

Please review your reference list to ensure that it is complete and correct. If you have cited papers that have been retracted, please include the rationale for doing so in the manuscript text, or remove these references and replace them with relevant current references. Any changes to the reference list should be mentioned in the rebuttal letter that accompanies your revised manuscript. If you need to cite a retracted article, indicate the article’s retracted status in the References list and also include a citation and full reference for the retraction notice.

[Note: HTML markup is below. Please do not edit.]

Reviewers' comments:

Reviewer's Responses to Questions

**Comments to the Author**

1. If the authors have adequately addressed your comments raised in a previous round of review and you feel that this manuscript is now acceptable for publication, you may indicate that here to bypass the “Comments to the Author” section, enter your conflict of interest statement in the “Confidential to Editor” section, and submit your "Accept" recommendation.

Reviewer #1: (No Response)

**********

2. Is the manuscript technically sound, and do the data support the conclusions?

The manuscript must describe a technically sound piece of scientific research with data that supports the conclusions. Experiments must have been conducted rigorously, with appropriate controls, replication, and sample sizes. The conclusions must be drawn appropriately based on the data presented.

Reviewer #1: Partly

**********

3. Has the statistical analysis been performed appropriately and rigorously?

Reviewer #1: Yes

**********

4. Have the authors made all data underlying the findings in their manuscript fully available?

The PLOS Data policy requires authors to make all data underlying the findings described in their manuscript fully available without restriction, with rare exception (please refer to the Data Availability Statement in the manuscript PDF file). The data should be provided as part of the manuscript or its supporting information, or deposited to a public repository. For example, in addition to summary statistics, the data points behind means, medians and variance measures should be available. If there are restrictions on publicly sharing data—e.g. participant privacy or use of data from a third party—those must be specified.

Reviewer #1: Yes

**********

5. Is the manuscript presented in an intelligible fashion and written in standard English?

PLOS ONE does not copyedit accepted manuscripts, so the language in submitted articles must be clear, correct, and unambiguous. Any typographical or grammatical errors should be corrected at revision, so please note any specific errors here.

Reviewer #1: Yes

**********

6. Review Comments to the Author

Please use the space provided to explain your answers to the questions above. You may also include additional comments for the author, including concerns about dual publication, research ethics, or publication ethics. (Please upload your review as an attachment if it exceeds 20,000 characters)

Reviewer #1: Thanks for the answers. However it is crucial to attend the next suggestions:

1. To facilitate reader analysis and interpretation, we recommend presenting the results as clearly as possible. Specifically, for the Immunofluorescence data, we suggest displaying all panels in color, (nor only the merge panels) as this will significantly improve the readability and assessment of the expression patterns. Standardize all the immunofluorescence images of the article in colors.

2. The authors use the term SNAIL and SNA1 (Fig. 4) as the same? Please clarify and standardize

**********

7. PLOS authors have the option to publish the peer review history of their article (what does this mean?). If published, this will include your full peer review and any attached files.

If you choose “no”, your identity will remain anonymous but your review may still be made public.

**Do you want your identity to be public for this peer review?**  For information about this choice, including consent withdrawal, please see our Privacy Policy.

Reviewer #1: No

**********

[NOTE: If reviewer comments were submitted as an attachment file, they will be attached to this email and accessible via the submission site. Please log into your account, locate the manuscript record, and check for the action link "View Attachments". If this link does not appear, there are no attachment files.]

To ensure your figures meet our technical requirements, please review our figure guidelines: https://journals.plos.org/plosone/s/figures

You may also use PLOS’s free figure tool, NAAS, to help you prepare publication quality figures: https://journals.plos.org/plosone/s/figures#loc-tools-for-figure-preparation.

NAAS will assess whether your figures meet our technical requirements by comparing each figure against our figure specifications.

# Author comment: Author response to Decision Letter 2

**DOI:** 10.1371/journal.pone.0354877.r004

13 Jul 2026

As requested, we have switched Figures 1, 2, S1 and S2 to color for panel standardization, and fixed the use of protein/gene names regarding Snail/SNA1 on Fig. 4 and in other selected places throughout the text.

Attachment Submitted filename: rebuttal to the reviewer R2.docx

# Editor report: Decision Letter 2

**DOI:** 10.1371/journal.pone.0354877.r005

**Authors:** Victoria Pando-Robles

15 Jul 2026

Molecular Analysis and Computational Modeling Reveal Temporally Separable Responses triggered by DENV-Induced Soluble Factors in Endothelial Cells

PONE-D-25-67733R2

Dear Dr. Vicente-Manzanares,

We’re pleased to inform you that your manuscript has been judged scientifically suitable for publication and will be formally accepted for publication once it meets all outstanding technical requirements.

Within one week, you’ll receive an e-mail detailing the required amendments. When these have been addressed, you’ll receive a formal acceptance letter and your manuscript will be scheduled for publication.

An invoice will be generated when your article is formally accepted. Please note, if your institution has a publishing partnership with PLOS and your article meets the relevant criteria, all or part of your publication costs will be covered. Please make sure your user information is up-to-date by logging into Editorial Manager at Editorial Manager® and clicking the ‘Update My Information' link at the top of the page. For questions related to billing, please contact billing support.

If your institution or institutions have a press office, please notify them about your upcoming paper to help maximize its impact. If they’ll be preparing press materials, please inform our press team as soon as possible -- no later than 48 hours after receiving the formal acceptance. Your manuscript will remain under strict press embargo until 2 pm Eastern Time on the date of publication. For more information, please contact onepress@plos.org.

Kind regards,

Victoria Pando-Robles, Ph.D.

Academic Editor

PLOS One

Additional Editor Comments (optional):

The authors attended all reviewer comments, the manuscript is ready to publishing

Reviewers' comments:

# Editor report: Acceptance letter

**DOI:** 10.1371/journal.pone.0354877.r006

**Authors:** Victoria Pando-Robles

PONE-D-25-67733R2

PLOS One

Dear Dr. Vicente-Manzanares,

I'm pleased to inform you that your manuscript has been deemed suitable for publication in PLOS One. Congratulations! Your manuscript is now being handed over to our production team.

At this stage, our production department will prepare your paper for publication. This includes ensuring the following:

* All references, tables, and figures are properly cited

* All relevant supporting information is included in the manuscript submission,

* There are no issues that prevent the paper from being properly typeset

You will receive further instructions from the production team, including instructions on how to review your proof when it is ready. Please keep in mind that we are working through a large volume of accepted articles, so please give us a few days to review your paper and let you know the next and final steps.

Lastly, if your institution or institutions have a press office, please let them know about your upcoming paper now to help maximize its impact. If they'll be preparing press materials, please inform our press team within the next 48 hours. Your manuscript will remain under strict press embargo until 2 pm Eastern Time on the date of publication. For more information, please contact onepress@plos.org.

You will receive an invoice from PLOS for your publication fee after your manuscript has reached the completed accept phase. If you receive an email requesting payment before acceptance or for any other service, this may be a phishing scheme. Learn how to identify phishing emails and protect your accounts at https://explore.plos.org/phishing.

If we can help with anything else, please email us at customercare@plos.org.

Thank you for submitting your work to PLOS ONE and supporting open access.

Kind regards,

PLOS ONE Editorial Office Staff

on behalf of

Victoria Pando-Robles

Academic Editor

PLOS One
